# Dynamics of contact behaviour by self-reported COVID-19 vaccination and infection status during the COVID-19 pandemic in Germany: an analysis of two large population-based studies

**DOI:** 10.1186/s12916-025-04211-x

**Published:** 2025-07-07

**Authors:** Lena Böff, Antonia Bartz, Manuela Harries, MuSPAD Consortium Group, Monika Strengert, Alex Dulovic, Nicole Schneiderhan-Marra, Stefanie Castell, Jana-Kristin Heise, Carolina Klett-Tammen, Gérard Krause, Pilar Hernandez, Daniela Gornyk, Monike Schlüter, Tobias Kerrines, Gerhard Bojara, Kerstin Frank, Knut Gubbe, Torsten Tonn, Oliver Kappert, Winfried V. Kern, Thomas Illig, Norman Klopp, Gottfried Roller, Michael Ziemons, COVIMOD Consortium Group, Tom Berger, Madhav Chaturvedi, Christopher I. Jarvis, Nicole Rübsamen, Stefan Scholz, Jasmin Walde, RESPINOW Consortium Group, Alex Dulovic, Nicole Schneiderhan-Marra, Carolina Klett-Tammen, Nils Bardeck, Wolfgang Bock, Michael Boehm, Laura-Inés Böhler, Johannes Bracher, Sebastian Contreras, Claudia Denkinger, Philipp Dönges, Cornelia Gottschick, Felix Guenther, Torben Heinsohn, Olga Hovardovska, Daniel Junker, Rolf Kaiser, Lisa Koeppel, Tyll Krueger, Alexander Kuhlmann, Patrick Marsall, Viola Priesemann, Ulrich Reinacher, Isti Rodiah, Melanie Schienle, Daniel Wolffram, André Karch, Annette Aigner, Veronika K. Jaeger, Berit Lange

**Affiliations:** 1 Department of Epidemiology, Helmholtz Centre for Infection Research (HZI), Braunschweig, Germany; 2 Institute of Epidemiology and Social Medicine, University of Münster, Münster, Germany; 3 Charité-Universitätsmedizin Berlin, Corporate Member of Freie Universität Berlin and Humboldt-Universität zu Berlin, Institute of Biometry and Clinical Epidemiology, Berlin, Germany; 4 Institute for Infectious Disease Epidemiology, TWINCORE, Hannover, Lower Saxony Germany

**Keywords:** SARS-CoV-2, Covid-19, Pandemic, Seroprevalence study, Social contact survey, Social contact behaviour, Immunity status, Vaccination status, Previous infections

## Abstract

**Background:**

Contact behaviour is crucial to assess and predict transmission of respiratory pathogens like SARS-CoV-2. Contact behaviour has traditionally been assessed in cross-sectional surveys and not as part of longitudinal population-based studies which simultaneously measure infection frequency and vaccination coverage. During the COVID-19 pandemic, several studies assessed contact behaviour over longer periods and correlated this to data on immunity. This can inform future dynamic modelling. Here, we assess how contact behaviour varied based on SARS-CoV-2 infection or vaccination status in two large population-based studies in Germany during 2021.

**Methods:**

We assessed direct encounters, separated into household and non-household contacts, in participants of MuSPAD (*n* = 12,641), a population-based cohort study, and COVIMOD (*n* = 31,260), a longitudinal contact survey. We calculated mean numbers of reported contacts and fitted negative binomial mixed-effects models to estimate the impact of immunity status, defined by vaccination or previous infection, on contact numbers; logistic mixed-effects models were used to examine the relationship between contact behaviour and seropositivity due to infection.

**Results:**

Contact numbers varied over the course of the pandemic from 7.6 to 10.8 per 24 h in MuSPAD and 2.1 to 3.1 per 24 h in COVIMOD. The number of non-household contacts was higher in participants who reported previous infections and vaccinations (contact ratio (CR) MuSPAD: 1.22 (95%CI 0.94–1.60); COVIMOD: 1.35 (CI 1.12–1.62)) compared to unvaccinated and uninfected individuals. Non-household contact numbers were also higher in fully vaccinated participants (MUSPAD: CR 1.15 (CI 1.05–1.26); COVIMOD: 1.43 (CI 1.32–1.56)) compared to unvaccinated individuals. Compared to individuals without household contacts, the odds for seropositivity due to infection were higher among MuSPAD individuals with three or more household contacts (odds ratio (OR) 1.54 (CI 1.12–2.13)) and eleven or more non-household contacts (OR 1.29 (CI 1.01–1.65)).

**Conclusions:**

Different contact behaviours based on infection and/or vaccination status suggest that public health policies targeting immunity status may influence the contact behaviour of those affected. A combined assessment of self-reported contacts, infections, and vaccinations as well as laboratory-confirmed serostatus in the population can support modelling of the spread of infections. This could help target containment policies and evaluate the impact of public health measures.

**Supplementary Information:**

The online version contains supplementary material available at 10.1186/s12916-025-04211-x.

## Background

Contact patterns in the human population are crucial in the transmission of most infectious diseases [1–3]. Empirical contact data resulting from scientific monitoring of social interactions is used to inform mathematical models to predict the dynamics of human-to-human infection spread [2] and help to anticipate the impact of control measures [4]. The COVID-19 pandemic highlights the importance of direct person-to-person contacts as a decisive factor in containment [5]. During the pandemic, interpersonal contact reduction measures were initiated by governments worldwide to reduce infectious disease burden [6]. In December 2020, COVID-19 vaccination campaigns began in Germany and initially prioritised the older, comorbid, and occupationally exposed population groups. In parallel to the advancing vaccination campaign in early 2021, the German government repeatedly prolonged the scaling down of public life [7]. In May 2021, with increasing vaccination coverage and declining incidence, contact restrictions were abolished for vaccinated and recently recovered individuals [8]. In June 2021, vaccination prioritisation was lifted [9] and in late August, the 2G and 3G rules were implemented, allowing vaccinated and recovered individuals (2G) plus individuals with a negative test result (3G) to gain entrance to additional aspects of public life, such as restaurants [10].

The population-based recording of contact behaviour is essential to evaluate the spread of the virus and to assess changes in its transmission related to variations in contact frequency [11]. Contact surveys traditionally ask specifically about the number, frequency, and degree of intimacy of contacts to assess regular contact behaviour of participants [12]. While pre-pandemic contact surveys such as POLYMOD were not very numerous and often only snapshots of short periods [2, 12–14], the continuous and large-scale contact surveys conducted during the pandemic, e.g. CoMix in Europe and COVIMOD in Germany [15–18], allow a more detailed understanding of how contact behaviour is actually distributed in the population. Furthermore, combining information on contact behaviour and how this varies in individuals with different vaccination and infection statuses enables us to better understand and estimate the potential effectiveness of vaccination strategies and non-pharmaceutical interventions, thus providing substantial value for public health policy. On the other hand, for future epidemic modelling efforts, intra-pandemic surveys have the disadvantage of being confounded by concurrent pandemic measures.

While contact surveys assess contact behaviour in a very detailed manner [14], typical seroepidemiologic studies have focused on quantifying previously unrecognized or unreported cases and have only assessed rough estimates of contact behaviour in a non-continuous way [19, 20]. Beyond what detailed contact surveys can do, these estimates provide a way to correlate information on contact frequency to population infection frequency, e.g. from seroprevalence estimations. This is why the comparison of information gathered on contacts in contact surveys and seroprevalence surveys during the pandemic is important to underlie modelling of infectious diseases of future epidemics and can also support learning to assess such real-time contact information more efficiently.

Here, we assess differences in social contact behaviour by self-reported SARS-CoV-2 infection and vaccination status as well as the association between laboratory-confirmed infection-based seropositivity and contact behaviour by comparing contact data from two population-based studies—one traditional contact survey and one seroprevalence study with some contact behaviour information—during the second year of the COVID-19 pandemic.

## Methods

### Study design

The study design and conduct of the MuSPAD study has been described previously [21]. In short, during 2020 and 2021, MuSPAD was established as a multicentre study assessing seroprevalence of antibodies against SARS-CoV-2 with consecutive cross-sectional studies at different locations throughout Germany. It has subsequently been redesigned as an adaptive population-based panel able to rapidly provide infection frequency and immunity estimates for different pathogens [22]. The presented analysis was performed in a subset of the MuSPAD population enrolled between January and August 2021 after the launch of the vaccination campaign (see Additional file 1: Fig. S3.1.1) [12, 18, 23–27]. Serological testing at the time of enrolment was done with a multiplex assay [28] which can distinguish between seropositivity due to infection or vaccination; furthermore, a self-administered questionnaire (online or paper-based) for completion at home collected information on contact behaviour (see Additional file 1: Table S1.1.1).

The COVIMOD study [18] is an observational, longitudinal study which collected self-reported data on person-to-person contacts as well as vaccination status and previous SARS-CoV-2 infections during the COVID-19 pandemic via online questionnaires. The market research company IPSOS recruited participants from members of the online panel i-say.com based on age, sex, and region quotas to ensure that the study population broadly matched the German population with regard to distribution of sociodemographic characteristics [23]. More details on the COVIMOD study can be found in the supplement (see Additional file 1: S1.2) and have also been described elsewhere [29].

### Variable definitions

A detailed overview of definitions of all variables can be found in Additional file 1: Table S2.0.1.

#### Definition of social contacts

Contacts in MuSPAD were defined as direct encounters involving a personal conversation (including encounters with recommended prevention measures, such as with a face mask or while maintaining a distance of 1.5 m [30]) or physical contact in the last 24 h [11]. In MuSPAD, we gathered data on the number of interactions by differentiating between contacts to specific household members (by age) and personal non-household contacts (aggregated into categories, e.g. colleagues, friends, clients) within the past 24 h (see Additional file 1: Table S1.1.1). Contacts in COVIMOD were defined as “people who you met in person and with whom you exchanged at least a few words, or with whom you had physical contact” in accordance with the definition used in the POLYMOD survey [12]. In COVIMOD, participants were asked to report each contact individually and were additionally able to report an aggregated number of contacts in a similar setting, so-called “group contacts” (grouped by age (under 18, 18 to 64, 65 and over) and location (school, work, other)). In both studies, household contacts are defined as contacts with members of a participant’s own household, while non-household contacts are contacts with people who do not belong to the participant’s household. The number of reported contacts in both studies might have been influenced by variations in the wording of contact questions and differences in response formats between the two studies.

In both studies, we truncated the reported number of contacts at 100 to minimise the impact of outliers on the mean number of contacts (see Additional file 1: S2).

#### Definition of vaccination status

In both studies, vaccination status (not vaccinated, partially vaccinated, fully vaccinated) was derived from the number of self-reported doses and vaccine type received. Participants who received one dose of the Janssen COVID-19 vaccine were considered as fully vaccinated, according to the guidelines at that time [31]. In both studies, participants who reported having received a first dose but provided no information on the vaccine type or a second dose were considered to be partially vaccinated. Entries for whom vaccination status was missing were eliminated from the analyses in COVIMOD (50 entries from 27 participants were removed) and were considered as unknown vaccination status in MuSPAD (*n* = 50) and examined in the group without reported vaccination in further analyses.

#### Definition of infection status

In MuSPAD, an individual’s knowledge about their own infection history was determined by self-reported positive test results (PCR or rapid antigen) and self-reported serology results. Positive self-reported serology results from 2020 and all positive infection tests were taken as confirmation of a past infection; positive self-reported serology results from 2021 were only used as confirmation of infection in unvaccinated individuals. In COVIMOD, infection status was established by combining self-reported data on current and past positive COVID-19 PCR and rapid antigen tests.

#### Definition of immunity status

In both studies, self-reported vaccination and infection history were then combined into one categorical immunity status variable reflecting the knowledge about the individual’s contact history with SARS-CoV-2 with ordinal levels expressing degrees of self-perceived protection against infection (no vaccination and no knowledge of infection, vaccination but no knowledge of infection, no vaccination but knowledge of infection, and vaccination and knowledge of infection).

#### Definition of serostatus

Serostatus was only available in the MuSPAD study. The serostatus variable was categorised based on laboratory results, thus revealing (undetected) infection or lack of seroconversion after vaccination and indicating potential protection. By identifying antibodies against spike- (S), receptor-binding domain- (RBD), and nucleocapsid- (NC) proteins, the multiplex assay allowed for the distinction between infection- or vaccine-acquired antibodies since vaccines do not contain an NC component. To account for more distant infections and the variable persistence of anti-NC with a possible serological picture similar to vaccination [32–34], the variable serostatus was corrected by the self-reported test history of participants (i.e. participants who reported a previous infection were considered to be seropositive). Similarly, participants with a constellation in the lab results indicating vaccination but who had no reported vaccination were classified as having had a previous infection. Based on this information, seropositivity due to infection was categorised as a binary variable to capture specifically infection-induced (NC) positive antibody status.

### Statistical analysis

Descriptive statistics comprised absolute and relative frequencies or mean, standard deviation (SD), median, interquartile range (IQR), minimum, and maximum for the respective variable scale. The time course of contact behaviour is displayed as mean numbers of contacts (weekly for MuSPAD and per survey wave for COVIMOD), with a locally weighted scatterplot smoothing (LOESS) estimate, a non-parametric variation of the linear least-squares regression to fit a curve [35]. Temporal trends of contact numbers were also stratified by immunity status and vaccination status.

We included a stringency index (ranging from 0 to 100) from the *Oxford Covid-19 Government Response Tracker* to help illustrate how strict government regulations were over time, with a higher stringency indicating stricter regulations [25].

With the help of Dagitty.net [27], directed acyclic graphs (DAGs) were built for the MuSPAD sample to determine the minimal adjustment sets of covariates for all regression models (see Additional file 1: S2.1). These same variables were also adapted for the COVIMOD regression models based on data availability.

Mixed-effects negative binomial regression models were used to assess the impact of self-reported immunity status and vaccination status on contact rates in the last 24 h for both studies [36]. Additionally, for the MuSPAD data, mixed-effects logistic regression models were used to assess the association between contact behaviour and seropositivity (NC antibodies) indicating infection. Given the two perspectives pursued in this study, contact behaviour appears as both an outcome as well as an exposure variable of the analyses. In the first context, the *reported* contact behaviour is seen as a consequence of the *known* immunity status, according to which individuals align their behaviour. In the second case, it is assumed that the contact behaviour collected in the study is representative of the regular contact pattern during the pandemic and thus serves as a proxy for prior contact behaviour, which in turn determines the risk of infection to which individuals are exposed and which is depicted in their *laboratory confirmed* serostatus. Separate models for all contact types were run in distinct parts of the sample (see Additional file 1: Table S2.1.1), and study site clusters were included as random intercepts in MuSPAD. In the COVIMOD models, the survey wave and federal state were included as random effects. Effect measures (incidence rate ratios for the negative binomial model, which we refer to as contact ratios (CR), and odds ratios (OR) for the logistic model) and confidence intervals (CI) were displayed in forest plots for MuSPAD and COVIMOD. All analyses were conducted using R version 4.1.2 [37], including the packages tidyverse [38], lme4 [36], and forestploter [39].

## Results

### Participant overview

Between 27 January and 17 August 2021, 12,641 MuSPAD participants with available contact information were recruited from 6 study locations at 7 collection periods, which corresponded to different moments in the pandemic with considerable variations in regional 7-day incidences and changes in government regulations (see Additional file 1: Fig. S4.0.1). In COVIMOD, 5694 participants generated a total of 31,260 responses over 17 survey waves (multiple counting for repeat participants across the waves) across Germany from 24 February to 31 December 2021.

The mean age is slightly higher in MuSPAD (54.0 vs 52.8), but in both MuSPAD and COVIMOD, 50% of participants are 55 years old or younger (Table 1). The proportion of women is higher in MuSPAD (59%) than in COVIMOD (48%). Contacts reported in each setting were higher in MuSPAD (mean contacts per 24 h: household 1.8, non-household 7.5) than in COVIMOD (0.7, 1.9). For a detailed presentation of descriptive summary statistics, refer to Additional file 1: Table S3.1.1.

**Table 1:** Summary characteristics of the MuSPAD and COVIMOD samples. Absolute and relative frequencies are given for categorical variables, minimum (min), maximum (max), mean (SD) and median (IQR) for continuous variables. Values are reported across all survey waves (i.e. COVIMOD participants who completed several waves appear more than once)

Attribute	MuSPAD	COVIMOD
Sample size: responses (participants)	12,641 (12,641)	31,260 (5694)
Analysis period	27 January to 17 August 2021	24 February to 31 December 2021
Study centres	Aachen, Osnabrück, Greifswald, Chemnitz, Magdeburg, Hanover	Germany-wide
Sex		
Female	7449 (58.9%)	15,016 (48.0%)
Male	5189 (41.1%)	16,177 (51.7%)
Diverse	<6	59 (0.2%)
Missing	-	8 (<0.1%)
Age		
Mean (SD)	54.0 (16.0)	52.8 (16.3)
Median (IQR)	55.0 (42.0–66.0)	55.0 (40.0–66.0)
Min | Max	18 | 99	18 | 92
Missing	38	261
Vaccination status		
No vaccination reported	7549 (59.7%)	9447 (30.2%)
Only one vaccine dose reported	2351 (18.6%)	4726 (15.1%)
Complete vaccination reported^a^	2691 (21.3%)	17,087 (54.7%)
Unknown vaccination status	50 (0.4%)	0 (0.0%)
Previous SARS-CoV-2 tests		
No test performed	5019 (39.7%)	17,001 (54.4%)
All performed SARS-CoV-2 tests were negative	6984 (55.3%)	12,573 (40.2%)
At least one positive SARS-CoV-2 test reported	590 (4.7%)	1251 (4.0%)
Invalid or missing response	48 (0.4%)	435 (1.4%)
Multiplex results in MuSPAD^b^		
No SARS-CoV- 2 antibodies	7568 (59.9%)	-
SARS-CoV-2 anti-S, anti-RBD	4426 (35.0%)	-
SARS-CoV-2 anti-NC, anti-S, anti-RBD	647 (5.1%)	-
Immunity status^c^		
No vaccination and no knowledge of infection	7112 (56.26%)	9100 (29.1%)
Vaccination but no knowledge of infection	4939 (39.07%)	20,909 (66.9%)
No vaccination but knowledge of infection	487 (3.85%)	347 (1.1%)
Vaccination and knowledge of infection	103 (0.81%)	904 (2.9%)
Total contacts		
Reported contacts in study population	117,514	82,884
Mean (SD) per person in 24h	9.3 (12.4)	2.7 (8.1)
Median (IQR) per person in 24h	5.0 (3.0–12.0)	1.0 (0.0–2.0)
Min | Max	0 | 100	0 | 302
Household contacts		
Reported contacts in study population	22,226	23,392
Mean (SD) per person in 24 h	1.8 (1.2)	0.7 (0.8)
Median (IQR) per person in 24 h	2.0 (1.0–2.0)	1.0 (0.0–1.0)
Min | Max	0 | 8	0 | 9
Non-household contacts		
Reported contacts in study population	95,396	59,492
Mean (SD) per person in 24 h	7.5 (12.4)	1.9 (8.1)
Median (IQR) per person in 24 h	3.0 (1.0–10.0)	0.0 (0.0–1.0)
Min | Max	0 | 100	0 | 300

^a^Single dose of the Janssen vaccine was considered as completely vaccinated

^b^Antibodies against spike (S), receptor-binding domain (RBD), or nucleocapsid (NC) protein

^c^Based on self-reported vaccinations and infections

### Vaccination status

**Fig. 1:**
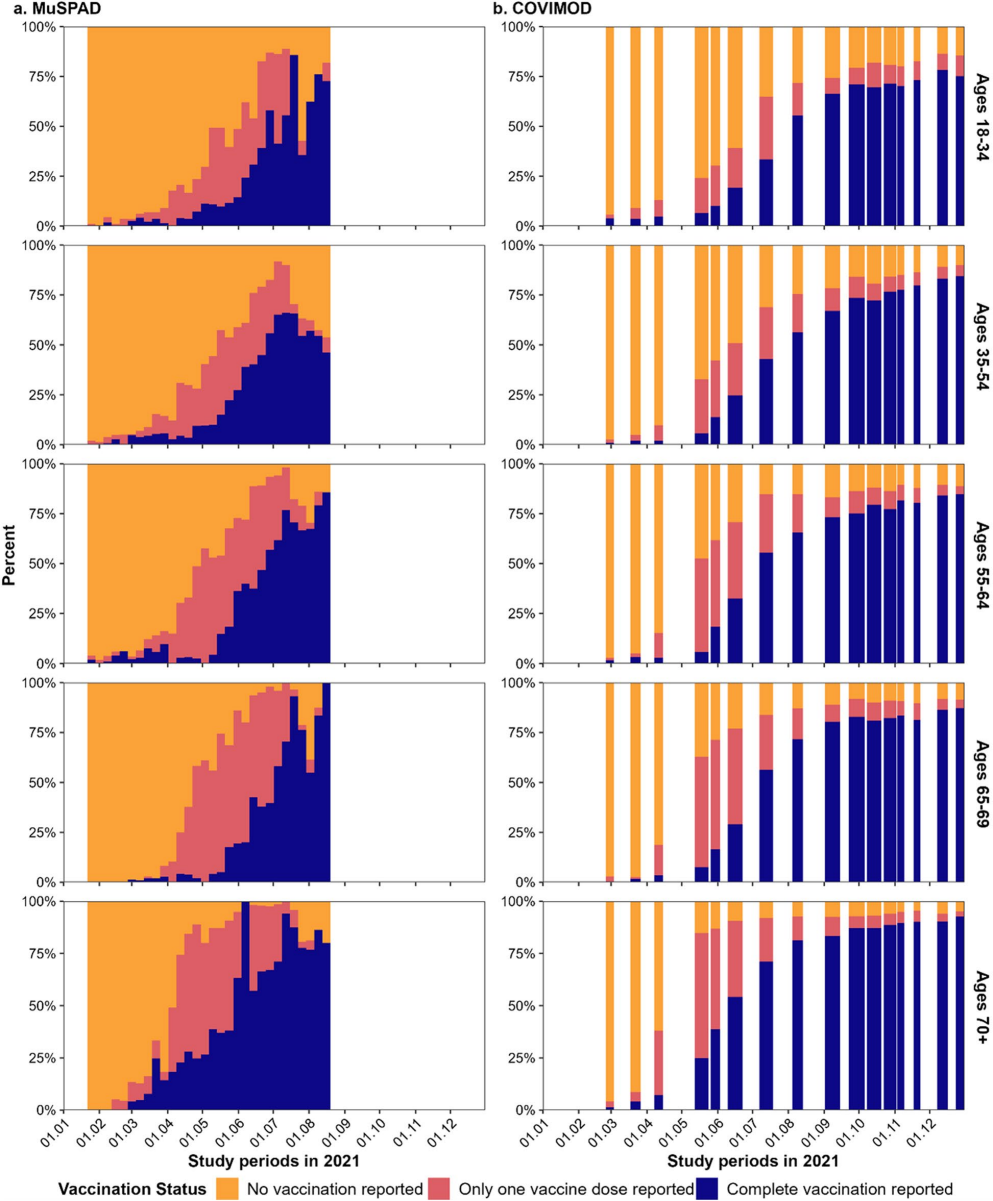
Vaccination status over time by age group and study in 2021 in the German population (MuSPAD & COVIMOD). Vaccination status is shown over time in 2021 and stratified by age groups for **a** MuSPAD and **b** COVIMOD. Individuals receiving the Janssen COVID-19 vaccine were counted as being fully vaccinated. Booster vaccines were not considered separately. For COVIMOD, the widths of the bars represent the length of each survey wave. Inconsistencies in vaccination rates over time in MuSPAD are likely reflections of study centres changing over the study period

While the two studies cover different periods and survey frequencies, they show similar patterns and complementary vaccination rates. In accordance with the vaccination strategy pursued in Germany, vaccination status generally increased over time across all age groups, with older age groups being vaccinated earlier and to a greater extent (Fig. 1). Because of recruitment in different phases of the vaccination campaign, about a fifth of MuSPAD participants each reported full (21%) or partial vaccination (19%), and nearly 60% reported no vaccination; in COVIMOD, 55% were fully vaccinated and only 15 and 30% were partially or not vaccinated, respectively (Table 1). When restricting both studies to the overlapping timeframe of 24 February to 17 August, vaccination rates were similar between the two studies: in MuSPAD, 25% were fully vaccinated, 21% partially vaccinated, and 54% not vaccinated; in COVIMOD, 26% were fully vaccinated, 24% partially vaccinated, and 51% not vaccinated (see Additional file 1: Table S3.2.1).

### Contact behaviour

#### Mean contacts

Overall mean contacts over 24 h ranged from 7.6 to 10.8 in the MuSPAD study (monthly mean contacts) and from 2.1 to 3.1 in COVIMOD (range of the mean contacts for the 16 survey waves). This was 6.6–13.0 (MuSPAD monthly mean contacts) and 2.1–4.0 (COVIMOD survey mean contacts) among fully vaccinated, and 5.7–9.5 (MuSPAD monthly mean contacts) and 1.7–3.4 (COVIMOD survey mean contacts) among not-vaccinated individuals. The tendency for MuSPAD participants to report more contacts than COVIMOD participants and for fully vaccinated participants to report more contacts than unvaccinated individuals remained when looking only at the timeframes when data were available for both studies (see Additional file 1: Table S3.2.1).

**Fig. 2:**
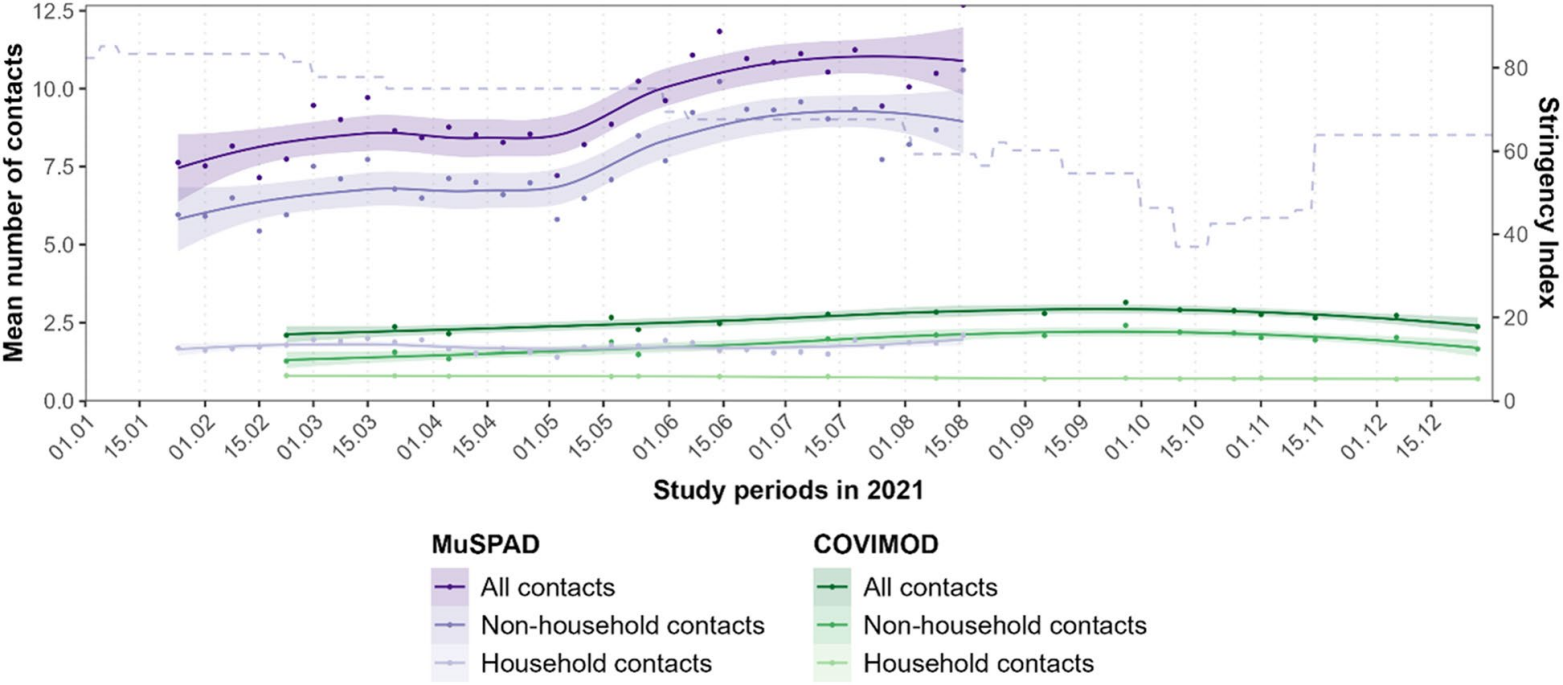
Contact numbers over time in 2021 in the German population. Temporal course of all, non-household, and household contacts in the MuSPAD and the COVIMOD study samples in 2021. Points mark mean number of contacts per week in MuSPAD and per survey wave in COVIMOD; smoothing to indicate temporal trends was done with the method “loess.” The dashed grey line illustrates the stringency index to quantify containment strategies implemented on the national level

The temporal course of contact behaviour over both studies showed rather constant values for household contacts. Total contacts and non-household contacts were markedly higher and altered with seasonal changes and adjustments in restriction measures (Fig. 2).

**Fig. 3:**
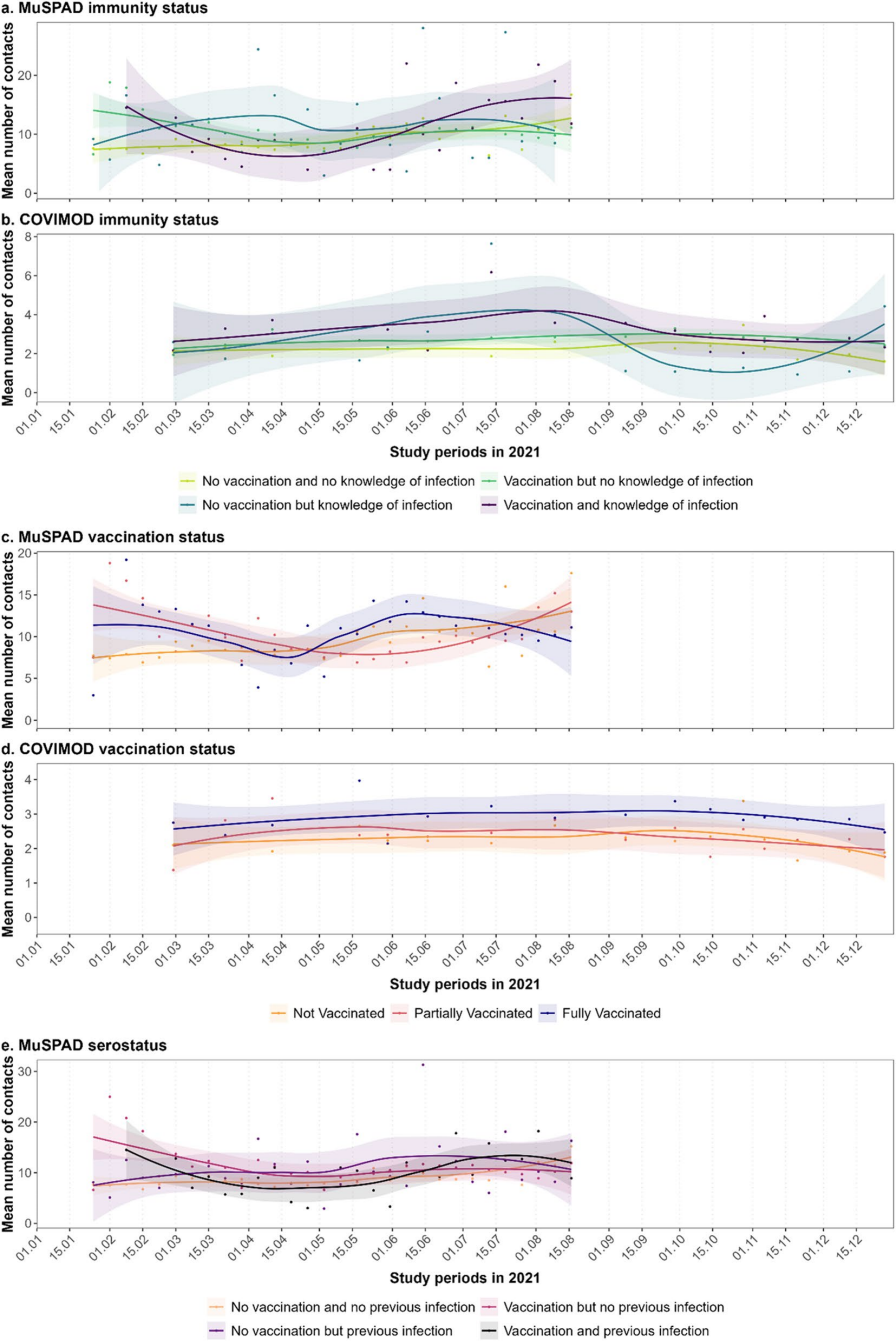
Mean number of contacts over time stratified by study, immunity status, vaccination status, and serostatus. The plots illustrate the mean number of overall contacts for **a** MuSPAD stratified by immunity status; **b** COVIMOD stratified by immunity status; **c** MuSPAD stratified by vaccination status; **d** COVIMOD stratified by vaccination status; **e** MuSPAD stratified by serostatus, based on laboratory results allowing the distinction between infection- or vaccine-acquired antibodies according to the detection of anti-NC, corrected by self-reported vaccination and test history. Points mark measured means; smoothing to indicate temporal trends was done with the method “loess.” The shaded regions indicate the 95% confidence interval of the estimates of the means derived with the loess smoothing method. For MuSPAD, contact behaviour was aggregated as weeks to account for varying contact behaviour on different days of the working week and weekend; for COVIMOD, the mean number of contacts was calculated by survey wave. Note differences in the *y*-axes between plots. A more detailed version of this plot including the stringency index can be found in Additional file 1: Figure S4.2.1

Contact behaviour stratified by immunity status, vaccination status, and serostatus demonstrates high variation over time (Fig. 3). In COVIMOD, fully vaccinated individuals consistently had more contacts than unvaccinated or partially vaccinated individuals (Fig. 3d). In MuSPAD, participants with knowledge of infection showed higher contact numbers for most of the study period (Fig. 3a); there is a similar pattern among individuals with serological evidence of infection (Fig. 3e). The groups without knowledge or laboratory evidence of infection or vaccination generally displayed lower contact numbers.

#### Number of contacts by immunity status and vaccination status

**Fig. 4:**
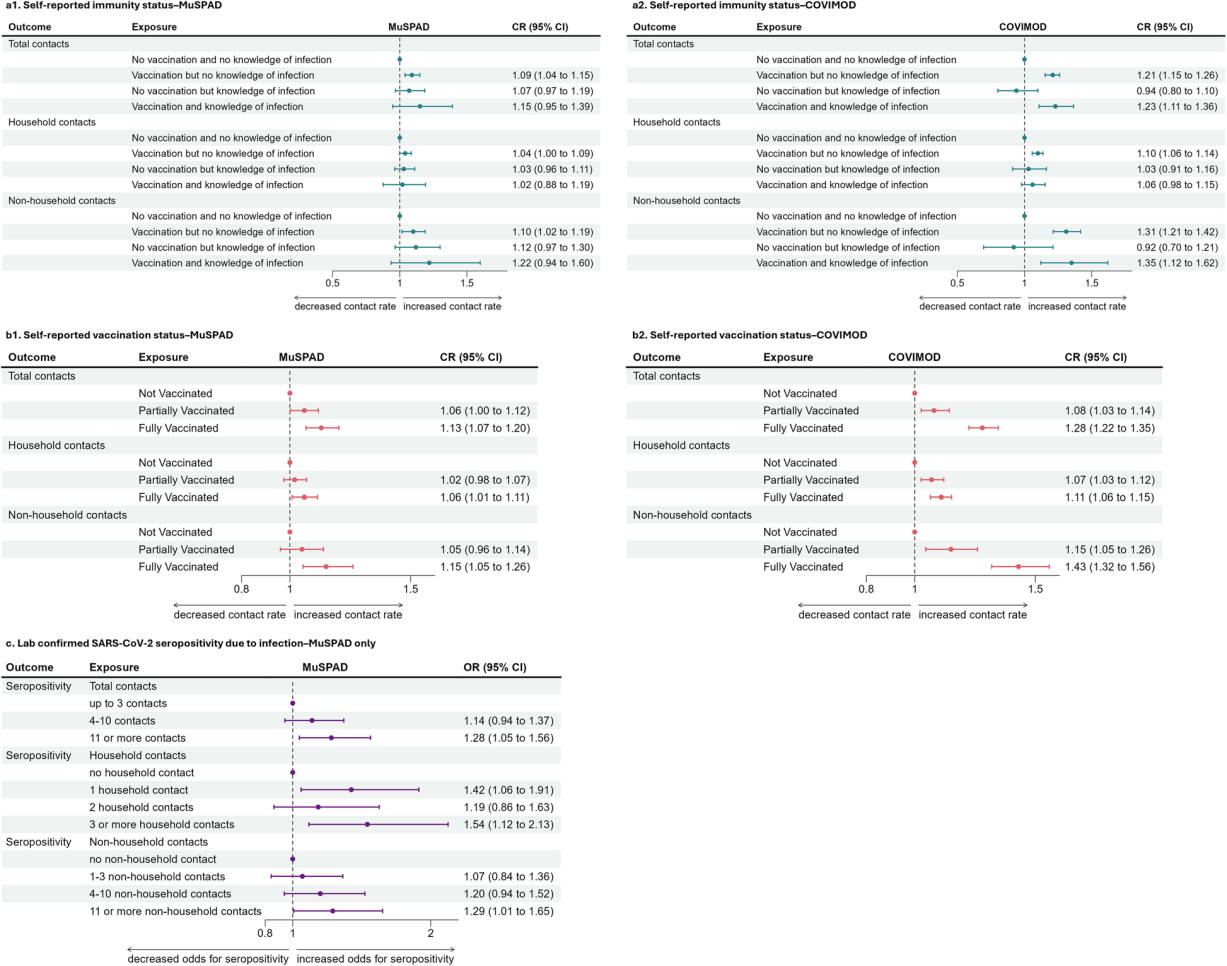
Forest plots of regression models, stratified by outcome, exposure, and study. Resulting effect estimates of regression models are displayed as **a** contact ratios (CRs) using negative binomial regression models for total, household, and non-household contact numbers by immunity status in **a1** MuSPAD and **a2** COVIMOD; **b** CRs using negative binomial regression models for contact numbers in different settings by vaccination status in **b1** MuSPAD and **b2** COVIMOD; and **c** odds ratios (ORs) for seropositivity due to infection (S>1, RBD>1, NC>1) using logistic regression models in MuSPAD. CRs for contacts and ORs for seropositivity are given with 95% confidence intervals (CI). For a list of adjusted variables for each model, refer to Additional file 1: Table S2.1.1

We found higher contact numbers outside the household in those participants with knowledge of vaccination or prior infection compared to the reference group with neither vaccination nor infection, though this was not the case for unvaccinated but previously infected individuals in COVIMOD. In MuSPAD, vaccinated but uninfected participants had 1.10 (CI 1.02–1.19) times as many non-household contacts as those without vaccination or infection, while individuals with both immunity status factors had a CR of 1.22 (CI 0.94–1.60) (Fig. 4a1). In COVIMOD, these estimates were 1.31 (CI 1.21–1.42) and 1.35 (CI 1.12–1.62), respectively (Fig. 4a2).

Partially vaccinated individuals in COVIMOD had 1.15 (CI 1.05–1.26) times as many non-household contacts as unvaccinated individuals, while fully vaccinated individuals had a CR of 1.43 (CI 1.32–1.56) (Fig. 4b2). Fully vaccinated individuals in MuSPAD also had more non-household contacts than unvaccinated individuals (CR of 1.15 (CI 1.05–1.26)), but the increase was less clear when looking at partially vaccinated individuals compared to unvaccinated individuals (CR of 1.05 (CI 0.96–1.14)) (Fig. 4b1).

We did not find a strong effect of knowledge of immunity on number of household contacts, with both studies having contact ratios close to and including one for household contacts regardless of immunity status (with the exception of COVIMOD participants reporting vaccination but no infection (CR 1.10 (CI 1.06–1.14))) (Fig. 4a).

#### Serostatus and number of contacts in MuSPAD

A higher number of total contacts was associated with increased odds of being seropositive; having 11 or more contacts had 1.28 times the odds of being seropositive compared to having up to 3 total contacts (CI 1.05–1.56; Fig. 4c). This was also observed when considering only non-household contacts (having 11 or more non-household contacts had an OR of 1.29 (CI 1.01–1.65) compared to having no non-household contacts) and only household contacts (OR of 1.54 (CI 1.12–2.13) for three or more household contacts compared to the reference group without household contacts). In the MuSPAD study, approximately 10% of participants reported no household contacts while around 20% indicated no non-household contacts. Among those with non-household contacts, the distribution was fairly even across groups, with roughly 20–30% of participants in each category, including around 24% who reported 11 or more non-household contacts (see Additional file 1: Table S3.3.1).

#### Influence of gender on contact behaviour and serostatus in MuSPAD

Stratifying the analysis by gender in the MuSPAD sample showed similar trends as in the overall study population (see Additional file 1: S5). However, descriptive statistics stratified by gender (see Additional file 1: Table S5.1.1) demonstrated that more women than men worked in healthcare and education, the two work sectors with the highest mean contact numbers. Women living with a child also had much higher odds for seropositivity than women in a household with no children, while the opposite was true for men (see Additional file 1: Figure S5.3.1). This was particularly pronounced among those living with children up to 5 years old (OR of 1.32 (CI 0.88–1.98) in women, 0.74 (CI 0.45–1.22) in men).

## Discussion

In the presented work, we integrate and compare results from two large population-based studies from Germany in 2021. Previous studies from various European countries have demonstrated that the number of contacts decreased during the COVID-19 pandemic compared to the years before the pandemic [15, 16, 18]. This study aimed to investigate whether contact behaviour varied explicitly by self-reported immunity status or vaccination status. We observed more contacts in those participants with self-reported vaccination or previous SARS-CoV-2 infection, and a higher number of non-household contacts was found among those with a serostatus indicative of a SARS-CoV-2 infection. While men and women had similar mean contact numbers overall and with respect to immunity status, women with children in the same household had a higher risk of infection, according to serological indicators, than those living without, an effect not seen in men.

Starting in mid-April 2021, partially and fully vaccinated individuals in COVIMOD had an increase in reported contacts compared to non-vaccinated individuals; this coincided with the start of the warm season and occurred near the end of Germany’s second lockdown, shortly before some restrictions were lifted [7]. An increase in contact numbers among vaccinated individuals was also observed in MuSPAD beginning in May 2021. This alignment of contact activities with the legal and seasonal context continued to be visible in the autumn period covered by COVIMOD; there was a higher number of contacts among fully vaccinated individuals compared to partially and not vaccinated individuals shortly after the implementation of the 3G and 2G rules, which loosened restrictions for vaccinated, recovered, and sometimes tested persons. Early access to vaccines was restricted to various subgroups of the population, initially targeting those most at risk for a severe COVID-19 disease course (e.g. the elderly) or individuals most at risk of coming into contact with infected individuals (e.g. healthcare workers). By about mid-June 2021, the vaccines were made available to a larger part of the population, until eventually all adults were eligible and able to access the vaccine. Therefore, increased contact among fully vaccinated individuals in the latter half of 2021 cannot be explained by vaccine availability. Overall, the increase in contacts among fully vaccinated individuals compared to unvaccinated individuals supports the idea that vaccination status impacts the number of contacts individuals have, an observation which was also seen in other European countries [40].

Looking at the immunity status, individuals who reported vaccinations with or without infections also had more contacts than individuals with no reported vaccinations or infections; in MuSPAD, this also applied to individuals with knowledge of infection but no vaccination. Understanding these nuances in contact behaviour by vaccination status and prior infections can be helpful to more accurately model transmission dynamics among different groups. In particular, the inclusion of such behavioural effects would be beneficial in the prediction of the impact of different vaccination strategies on potential burden of disease in ongoing epidemics. This is supported by a previous analysis from COVIMOD showing that contact frequency during the pandemic depended on whether participants belonged to high or low risk groups for severe disease course [29].

In MuSPAD, participants with a higher average number of contacts also had higher odds of seropositivity for SARS-CoV-2 anti-NC antibodies; however, in this study, we did not assess frequency of contacts prior to being seropositive. Therefore, in principle this could also reflect the effect of knowledge of previous infection on current contact behaviour. In a sensitivity analysis looking at contact behaviour based on seropositivity together with self-reported knowledge of infection, we found that participants with laboratory-confirmed seropositivity who also reported a previous infection had 1.14 (CI 0.99–1.30) times as many non-household contacts as participants with no laboratory-confirmed infection and no reported infection; on the other hand, individuals with laboratory-confirmed seropositivity but no reported previous infection had 0.91 (CI 0.78–1.06) times as many non-household contacts as participants with no laboratory-confirmed infection and no reported infection (see Additional file 1: S6). We believe that this warrants further assessment when longitudinal assessments become available (with surveys conducted in 2024). Additionally, a survey design also capturing motivational aspects of contact behaviour informed by the results of a possible qualitative approach to characterise the effect of an individual’s immunity status on contact decisions could provide further insight here in the future and help clarify causal links between knowledge of immunity status, contact behaviour, and seroepidemiological status of an individual. The pronounced higher odds of seropositivity for individuals with household contacts compared to those living alone underlines the importance of the living environment in transmission risk, where isolation is difficult, although direct causality cannot be established [41].

### Strengths and limitations

Drawing conclusions from the comparison of the two distinctly designed and organised studies has clear limitations in terms of different source populations (population registry in MuSPAD; online panel in COVIMOD), different data entry methods (only partly online in MuSPAD; fully online in COVIMOD), different data collection methods (non-household contacts being reported in categories in MuSPAD versus individually in COVIMOD), and different household structures (only 16% of MuSPAD participants lived in single-person households, compared to 36% in COVIMOD, see Additional file 1: Table S3.1.1). We found a relatively large difference in the absolute numbers of contacts, with MuSPAD participants reporting more contacts than COVIMOD participants overall. This is likely due to differences in the questionnaire design and the collection of contact data despite having used adapted shortened data collection instruments from COVIMOD early on in MuSPAD [42]. Differences in the wording of contact questions and the offered response formats might have introduced systematic variation in how contacts were reported in both studies. The reporting of non-household contacts in a categorical format as aggregated numbers in MuSPAD might have led to rounding or over-reporting tendencies. On the other hand, the individual reporting structure in COVIMOD might have contributed to under-reporting due to participant fatigue or recall bias. Furthermore, the observed discrepancies in household size between the two cohorts could explain part of the differences in reported contacts.

An additional limitation is that household contacts, which were supposed to imply contact persons actually living in the same household, had several issues with misclassification. Since participants in MuSPAD who reported living alone revealed average household contact numbers above 0, the question was presumably understood differently by at least some of the participants who classified contacts with non-household members as household contacts. Furthermore, MuSPAD participants were asked to report household contacts by their relation (e.g. sister, brother) but were only able to report one contact per category. Therefore, the number of household contacts is likely an underestimate of the true value.

We have directed the analysis towards a causal approach, thereby aiming at the mandate for observational research [43]. However, the simultaneous collection of laboratory and questionnaire data in MuSPAD, without assessment of modifications in contact behaviour over the course of the pandemic, makes it difficult to draw a definite causal conclusion about the temporal sequence of vaccination, infection, and contacts. However, since information on self-reported vaccination and infection was collected at the same time as information on contacts, we can assume that participants’ self-reported vaccination and infection generally predated their contacts, since their assumed vaccination/infection status would have been mostly unchanged during the 24-h time frame of interest for which contact data were collected. In the same context, there is some caveat when comparing contact behaviour over time for MuSPAD, as the MuSPAD data were based on repeated cross-sectional studies of different individuals and did not reflect the behaviour of a longitudinally followed cohort. It is particularly problematic that age groups showed different contact behaviour but represented different proportions of each immunity status stratum throughout the study period.

One of the limitations specific to COVIMOD concerned data collection. Some repeat participants had different individuals fill out the survey in new waves, meaning that an analysis of behavioural differences may be inaccurate since an individual’s vaccination status was determined based on answers to current and previous survey waves. This issue was only present in a minority of cases but could bias the results towards null since actual behavioural differences by vaccination status would be masked by incorrectly classified vaccination status. Another limitation comes from COVIMOD being based on an online panel since this raises questions about overall representativeness of the general population, in particular for individuals with less access to the internet.

Analyses based on self-reporting usually overestimate compliance to protective measures by a wide margin compared to observed adherence, and the use of observation methods is recommended [44]. For the information on contact behaviour, a form of social desirability bias might have applied, so future studies using approaches like direct observation could validate our findings. Nonetheless, in view of the difficulty of observational methods for individual contact behaviour, the concise assessment of contact behaviour used here might at least have mitigated a recall bias.

We believe that with these limitations in mind, relative comparisons—as done in this study—can still be drawn. In future analyses, it would be interesting to quantify the impact of differences in study design methodology and data collection on the estimation of the absolute numbers of contacts, e.g. for different contact settings. This would allow us to more easily integrate findings from different studies, e.g. for dynamic modelling.

The sample sizes of 12,641 participants with a total of 117,514 recorded contacts in MuSPAD and 31,260 entries (from 5,694 individual participants) reporting 82,884 contacts in COVIMOD are to be taken as a strength. In addition, the multi-site approach with random sampling and the extended collection period contributed to a relatively comprehensive recording of contact patterns throughout the German population. By combining data from two studies, we also gain insights into how different approaches in study design yield comparable results. The simultaneous recording of contact behaviour and data on seroprevalence can yield a benefit in informing discussions on infection dynamics and suitable restriction measures.

The use of study populations that aim to be mostly representative for the German population, paired with the finding that both independent studies have similar effects, supports the robustness of the study results and their potential generalisability to the entire German population. However, the study was restricted to individuals aged 18 and over since minors in Germany received a vaccine recommendation much later than adults. Generalisability to other countries and future pandemics may be limited, but since COVIMOD’s questionnaire was based on CoMix—a social contact study conducted during the COVID-19 pandemic in 20 European countries—and served as a basis for MuSPAD’s contact questions, we are better able to compare our results to findings in other European countries [15, 16]. This could also make it easier to compare different government approaches in the future to gain a better understanding of the impact of different regulations on contact behaviour.

## Conclusions

We found an increased number of contacts among vaccinated individuals compared to unvaccinated individuals. This indicates that vaccination status may influence behaviour. The relationship between combined vaccination and infection status and contact numbers is less clear. In addition, seropositivity due to infection was more common among individuals with more household contacts and among women living with children. The inclusion of behavioural differences in the prediction of the effect of vaccination strategies on potential burden of disease in ongoing epidemics would be beneficial. The real-time joint collection of contact behaviour, self-reported immunity status, and laboratory-based serostatus can be used to align contact dynamics.

## Supplementary Information

Additional File 1: Tables S1.1.1-S6.0.1. TableS1.1.1 – MuSPAD study questionnaire. TableS1.2.1 – COVIMOD study questionnaire. TableS2.0.1 – Variable definitions. TableS2.1.1 – Adjustment variable sets. TableS3.1.1 – Demographic characteristics. TableS3.2.1 – Demographic characteristics in restricted timeframe. TableS3.3.1 – MuSPAD contact groups. TableS5.1.1 – MuSPAD demographics by gender. TablsS5.2.1 – MuSPAD gender-stratified adjustment sets. TablsS5.3.1 – MuSPAD gender-stratified household child adjustment sets. TableS6.0.1 – MuSPAD sensitivity analysis adjust sets. Figures S2.1.1-S6.0.1. FigS2.1.1 – DAG for immunity status and household contacts. FigS2.1.2 – DAG for immunity status and non-household contacts. FigS2.1.3 – DAG for household contacts and seropositivity. FigS2.1.4 – DAG for non-household contacts and seropositivity. FigS2.1.5 – DAG for household children and seropositivity. FigS3.1.1 – MuSPAD study population. FigS3.1.2 – COVIMOD inclusion and exclusion. FigS4.0.1 – MuSPAD collection period timings. FigS4.1.1 – Contacts by immunity status boxplot. FigS4.1.2 – Contacts by vaccination status boxplot. FigS4.1.3 – Contacts by serostatus boxplot. FigS4.2.1 – Mean contacts over time. FigS5.2.1 – MuSPAD seropositivity by gender. FigS5.3.1 – MuSPAD seropositivity by household child and gender. FigS6.0.1 – MuSPAD contacts by serostatus and knowledge thereof.

## Data Availability

The datasets used and analysed during the current study are available from the corresponding author on reasonable request. The data for the stringency index that support the findings of this study are available from the Oxford Covid-19 Government Response Tracker [25]. The data on national and regional 7-day incidence rates during the Covid-19 pandemic are available from the Robert Koch Institute [26]. Regarding data from the NAKO study, Hannover researchers have the opportunity to apply for data usage in accordance with the official regulations and specifications. For more detailed information, please visit https://transfer.nako.de.

## Abbreviations

2G: German COVID-19 restriction requiring vaccination or recent recovery
3G: German COVID-19 restriction requiring vaccination, recent recovery, or negative test
CI: Confidence interval
CoMix: A group of studies on contact behaviour during the COVID-19 pandemic conducted in several European countries
COVID-19: Coronavirus disease caused by SARS-CoV-2
COVIMOD: A German study on contact behaviour during the COVID-19 pandemic
CR: Contact ratio
DAG: Directed acyclic graph
IPSOS: A market research company
MuSPAD: Multilokale und Serielle Prävalenzstudie zu Antikörpern gegen (respiratorische) Infektionserkrankungen Deutschland; a German seroprevalence study that included contact data
NC: Nucleocapsid
NPIs: Non-pharmaceutical interventions (e.g. contact reduction, mask wearing)
OR: Odds ratio
POLYMOD: A landmark study on contact behaviour
RBD: Receptor-binding domain
RKI: Robert Koch Institut (Germany’s federal public health agency)
S: Spike
SARS-CoV-2: Severe acute respiratory syndrome coronavirus 2
SD: Standard deviation
