# Supplementary material for: Dynamics of contact behaviour by self-reported COVID-19 vaccination and infection status during the COVID-19 pandemic in Germany: an analysis of two large population-based studies
Source: BMC Med. 2025 Jul 7;23:406. doi: 10.1186/s12916-025-04211-x (PMC12235905; doi:10.1186/s12916-025-04211-x)
Supplement: Supplementary file 1 — Additional File 1: Tables S1.1.1-S6.0.1. TableS1.1.1 – MuSPAD study questionnaire. TableS1.2.1 – COVIMOD study questionnaire. TableS2.0.1 – Variable definitions. TableS2.1.1 – Adjustment variable sets. TableS3.1.1 – Demographic characteristics. TableS3.2.1 – Demographic characteristics in restricted timeframe. TableS3.3.1 – MuSPAD contact groups. TableS5.1.1 – MuSPAD demographics by gender. TablsS5.2.1 – MuSPAD gender-stratified adjustment sets. TablsS5.3.1 – MuSPAD gender-stratified household child adjustment sets. TableS6.0.1 – MuSPAD sensitivity analysis adjust sets. Figures S2.1.1-S6.0.1. FigS2.1.1 – DAG for immunity status and household contacts. FigS2.1.2 – DAG for immunity status and non-household contacts. FigS2.1.3 – DAG for household contacts and seropositivity. FigS2.1.4 – DAG for non-household contacts and seropositivity. FigS2.1.5 – DAG for household children and seropositivity. FigS3.1.1 – MuSPAD study population. FigS3.1.2 – COVIMOD inclusion and exclusion. FigS4.0.1 – MuSPAD collection period timings. FigS4.1.1 – Contacts by immunity status boxplot. FigS4.1.2 – Contacts by vaccination status boxplot. FigS4.1.3 – Contacts by serostatus boxplot. FigS4.2.1 – Mean contacts over time. FigS5.2.1 – MuSPAD seropositivity by gender. FigS5.3.1 – MuSPAD seropositivity by household child and gender. FigS6.0.1 – MuSPAD contacts by serostatus and knowledge thereof. [file 12916_2025_4211_MOESM1_ESM.pdf]

Dynamics of contact behaviour by self-reported COVID-19 vaccination and infection status during the COVID-19 pandemic in Germany: An analysis of two large population-based studies

Additional file 1

Lena Böff[1]#, Antonia Bartz[2]#, Manuela Harries[1], MuSPAD Consortium Group, COVIMOD Consortium Group, RESPINOW Consortium Group, André Karch[2], Annette Aigner[3]#, Veronika K Jaeger[2]*#, Berit Lange[1,4]*#

Affiliations

1 Department of Epidemiology, Helmholtz Centre for Infection Research (HZI), Braunschweig, Germany

2 Institute of Epidemiology and Social Medicine, University of Münster, Münster, Germany

3 Charité-Universitätsmedizin Berlin, Corporate Member of Freie Universität Berlin and Humboldt-Universität zu Berlin, Institute of Biometry and Clinical Epidemiology, Berlin, Berlin, Germany

4 Institute for Infectious Disease Epidemiology, TWINCORE, Hannover, Lower Saxony, Germany

*Corresponding authors (veronika.jaeger@ukmuenster.de; berit.lange@helmholtz-hzi.de)
#Equal contributors

Contents

S1 Study Designs 5

S1.1 MuSPAD 5

Table S1.1.1 Questions on vaccination, infections, and contact behaviour from the MuSPAD study questionnaire. 5

S1.2 COVIMOD 7

Table S1.2.1 Questions on vaccination status from the COVIMOD study questionnaire. 8

S2 Variables 9

Table S2.0.1 Definitions of variables for MuSPAD and COVIMOD. 10

S2.1 DAGs and adjustment sets 12

Figure S2.1.1 DAG for the impact of assumed immunity status on household contacts. 12

Figure S2.1.2 DAG for the impact of assumed immunity status on non-household contacts. 12

Figure S2.1.3 DAG for the impact of household contacts on seropositivity due to infection. 13

Figure S2.1.4 DAG for the impact of non-household contacts on seropositivity due to infection. 13

Figure S2.1.5 DAG for the impact of living with a child in the household on seropositivity due to infection. 14

Table S2.1.1 Adjusted variables in regression models for MuSPAD and COVIMOD. 14

S3 Demographics 16

S3.1 Study population 16

Figure S3.1.1 Flow chart of the MuSPAD study population. 16

Figure S3.1.2 Inclusion and exclusion of COVIMOD survey entries. 17

Table S3.1.1 Demographic characteristics of the MuSPAD and COVIMOD studies. 18

S3.2 Restricted timeframe 21

Table S3.2.1 Summary characteristics of the MuSPAD and COVIMOD samples for restricted collection period form 24 February to 17 August, 2021. 21
S3.3 Contact groups in MuSPAD........ 22
Table S3.3.1 Contact groups of the MuSPAD sample. 22
S4 Contact behaviour over time 23
Figure S4.0.1 Timing of MuSPAD collection periods by study centre......... 23
S4.1 Boxplots 24
Figure S4.1.1 Contact numbers stratified by immunity status......... 25
Figure S4.1.2 Contact numbers stratified by vaccination status......... 26
Figure S4.1.3 Contact numbers stratified by serostatus......... 27
S4.2 Mean Contact Plots........ 28
Figure S4.2.1 Mean number of contacts over time stratified by study, immunity status, vaccination status, and serostatus. 28
S5 Sex-specific subgroup analyses (MuSPAD only) 30
S5.1 Summary characteristics by female and male subsamples........ 30
Table S5.1.1 Summary demographics of the study population, stratified by females and males. 30
S5.2 Effect of seropositivity on contact numbers 32
Figure S5.2.1 Odds ratios for seropositivity due to infection in the MuSPAD gender subgroups. 32
Table S5.2.1 Adjusted variables in gender-stratified logistic regression models for MuSPAD......... 33
S5.3 Effect of children in the household on seropositivity 34
Figure S5.3.1 Odds ratios for seropositivity due to infection when living with a child in the MuSPAD gender subgroups. 34
Table S5.3.1 Adjusted variables in gender-stratified logistic regression models for child in household for MuSPAD......... 34
S6 Serostatus and knowledge thereof........ 35
Figure S6.0.1 Forest plot of contact ratios by seropositivity and knowledge thereof. 35

Table S6.0.1 Adjusted variables in sensitivity analysis regression models for MuSPAD. 36

S1 Study Designs

S1.1 MuSPAD

Table S1.1.1 Questions on vaccination, infections, and contact behaviour from the MuSPAD study questionnaire.

Vaccination

3.2 Did you receive the first dose of the COVID-19 vaccination?
☐ Yes ☐ No ☐ I do not know ☐ Not stated

3.2.1 If yes, when did you receive the first dose? |__|__|. |__|__|.202|__| ☐ I do not know ☐ Not stated

3.2.2 Which vaccine have you been vaccinated with?
☐ BioNTech/Pfizer BNT162b2 (COMIRNATY)
☐ Moderna mRNA-1273
☐ Gamaleya Sputnik V
☐ Oxford/AstraZeneca AZD1222
☐ Sinopharm BBIBP-CorV
☐ Serum Institute of India Covishield
☐ Bharat Biotech Covaxin
☐ Other
☐ I do not know
☐ Not stated

3.2.3 If „other“, which one? ________________________

3.2.4 If available, please enter batch number_____________________

3.2.5 Did you receive the second dose of the COVID-19 vaccination?

☐ Yes ☐ No ☐ I do not know ☐ Not stated

3.2.4 If yes, when did you receive the second dose? |__|__|. |__|__|.202|__| ☐ I do not know ☐ Not stated

3.2.5 Which vaccine have you been vaccinated with?
☐ BioNTech/Pfizer BNT162b2 (COMIRNATY)
☐ Moderna mRNA-1273
☐ Gamaleya Sputnik V
☐ Oxford/AstraZeneca AZD1222
☐ Sinopharm BBIBP-CorV
☐ Serum Institute of India Covishield
☐ Bharat Biotech Covaxin
☐ Other
☐ I do not know
☐ Not stated

3.2.6 If „other“, which one? ________________________

3.2.7 If available, please enter batch number_____________________

Infection

14. Have you ever been tested for SARS-CoV-2 (new coronavirus) since 01st February 2020?

For this laboratory test, a sample (throat swab) is obtained through the mouth or nose, sputum (medical term for phlegm) or throat lavage water.

☐ **Yes** ☐ **No** ☐ I do not know ☐ Not stated

14a. If yes, which statement is true?

☐ **I had at least one positive test result**

Please enter the date of the first positive test: |__|__|. |__|__|.202|__|

☐ I do not know ☐ Not stated

☐ **I had only negative test results**

Please enter the date of the last negative test: |__|__|. |__|__|.202|__|

☐ I do not know ☐ Not stated

☐ **I do not know my test result**

14.1 Have you ever been tested for antibodies against SARS-CoV-2 (new coronavirus) since 1st February 2020? *A blood sample is drawn for this laboratory test.*

☐ **Yes** ☐ **No** ☐ I do not know ☐ Not stated

14.1a If yes, which statement is true?

☐ **I had at least one positive test result** (continue with question 14b)

→ Please enter the date of the first positive test: |__|__|. |__|__|.202|__|

☐ I do not know

☐ Not stated

☐ **I had only negative test results** (continue with question 15)

→ Please enter the date of the last negative test: |__|__|. |__|__|.202|__|

☐ I do not know

☐ Not stated

☐ **I do not know my test result**

Contact behaviour

13. With which of the following people **within** your household did you have direct personal contact between 5 a.m. yesterday and 5 a.m. today?

			How old is the person?
Mother	☐ Yes	☐ No	Years
Father	☐ Yes	☐ No	Years
Partner	☐ Yes	☐ No	Years
Grandmother	☐ Yes	☐ No	Years
Grandfather	☐ Yes	☐ No	Years
Sister	☐ Yes	☐ No	Years
Brother	☐ Yes	☐ No	Years
Daughter	☐ Yes	☐ No	Years
Son	☐ Yes	☐ No	Years
Flatmate	☐ Yes	☐ No	Years
Other	☐ Yes	☐ No	Years

14. And with which other persons **outside** the household did you have direct personal contact between 5 a.m. yesterday and 5 a.m. today? Please also include persons with whom you had a conversation at a distance of 1.5 metres, wearing a mask or otherwise socially distanced.

a. Friends	☐ Yes	☐ No	Number of contacts:
b. Relatives	☐ Yes	☐ No	Number of contacts:
c. Colleagues	☐ Yes	☐ No	Number of contacts:
d. People you spoke to in shops	☐ Yes	☐ No	Number of contacts:
e. Teacher	☐ Yes	☐ No	Number of contacts:
f. Classmates/fellow students	☐ Yes	☐ No	Number of contacts:
g. Transport person e.g. taxi driver, bus/train driver	☐ Yes	☐ No	Number of contacts:
h. Health services personnel e.g. doctor / dentist	☐ Yes	☐ No	Number of contacts:
i. Cleaner	☐ Yes	☐ No	Number of contacts:
j. Caregiver	☐ Yes	☐ No	Number of contacts:
k. People in a place of worship	☐ Yes	☐ No	Number of contacts:
l. Customers/clients	☐ Yes	☐ No	Number of contacts:
m. Other	☐ Yes	☐ No	Number of contacts:
n. Nobody	☐ Yes	☐ No	

S1.2 COVIMOD

The COVIMOD contact survey was a primary, observational, repeated cross-sectional study that collected data on person-to-person contacts during the COVID-19 pandemic via online questionnaires. Although we mostly considered each wave as an individual cross-sectional study, participants were repeatedly contacted to participate in numerous waves of the survey, thus providing some longitudinal data as well. New participants were also recruited in each survey wave to compensate for declining participant numbers; the overall sample size was restricted based on available funding. The market research company Ipsos-MORI was commissioned to recruit participants via their online panel on i-say.com [23]. Participants were recruited based on quotas for federal state, sex, and age representative of the German population. In addition, some parents were asked to fill out the survey as a proxy for their child instead of themselves, leading to a potential underrepresentation of middle-aged persons with children in the participants.

The questionnaire was based on the questionnaire of the CoMix study, a multi-country European study on contacts during the COVID-19 pandemic [24]. In addition to demographic information, the COVIMOD survey collected data on social contacts, current behaviours, and attitudes towards COVID-19. Participants were asked to recall all social contacts from 5am the previous day until 5am on the day of the survey. A contact was defined as “people who you met in person and with whom you exchanged at least a few words, or with whom you had physical contact” in accordance with the definition used in the POLYMOD Survey, a landmark study on contact behaviour in Germany and seven other European countries that was run prior to the pandemic in 2005 and 2006 [12]. Additional data on these contacts, including age and sex of the contact, relation to the contact, and contact duration and location, were also collected. The study questionnaire is published elsewhere [18] and the vaccination questions, not yet available at the time the questionnaire was published, can be found in Table 1.2.1.

The first wave of the study began on 30 April 2020 and the final survey wave ended on 31 December 2021; the 33 conducted survey waves were each spaced approximately one to five weeks apart. Beginning in survey wave 3, participants were able to report an aggregated number of contacts in a similar setting if there were too many contacts to list them all individually; moving forward, these will be referred to as group contacts. These contacts were grouped by location (school, work, other) and age group (under 18, 18 to 64, 65 and over). Beginning in January 2021 (survey wave 17), questions on COVID-19 vaccinations were also included; these questions asked for the number of doses received, the dates of each dose, and the names of the vaccines. Since under 2% of participants were partially or fully vaccinated during survey wave 17, this study is restricted to survey waves 18 to 33. Individuals

under the age of 18 were also removed from this study since they only became eligible for the vaccinations during later survey waves. Exact dates of the survey waves and respective sample sizes are available in Table 3.1.1.

Table S1.2.1 Questions on vaccination status from the COVIMOD study questionnaire.

QXX1. Have you been vaccinated against the virus that causes Coronavirus (COVID-19)?	1. Yes<br>2. No
QXX2. How many doses of vaccinations have you had against the virus that causes Coronavirus (COVID-19)?	WRITE IN NUMBER OF DOSES: [RANGE FROM 1 TO 2 (IN LATER WAVES, UP TO 3)]
QXX3. Please provide the dates you received your vaccination(s) against the virus that causes COVID-19.	INSERT NUMBER OF DATE BOXES THAT MATCHES ANSWER AT QXX2. FUTURE DATES NOT PERMITTED<br>Don't know
QXX4. What vaccine did you receive?	1.Comirnaty (Biontech/Pfizer)<br>2.COVID-19 Vaccine Moderna (Moderna)<br>4.Vaxzevria (AstraZeneca)<br>5. COVID-19 Vaccine Jansen (Johnson & Johnson)<br>3.Don't know
QZZ1. You previously said you have been vaccinated against the virus that causes Coronavirus (COVID-19). Have you had any new doses of the vaccination since you last completed the survey?	1. Yes<br>2. No
QZZ2. How many new doses of vaccinations have you had against the virus that causes Coronavirus (COVID-19)?	WRITE IN NUMBER OF DOSES: [RANGE FROM 1 TO 2 (IN LATER WAVES, UP TO 3)]
QZZ3. Please provide the dates you received your new vaccination(s) against the virus that causes COVID-19.	INSERT NUMBER OF DATE BOXES THAT MATCHES ANSWER AT QZZ2. FUTURE DATES NOT PERMITTED<br>Don't know
QZZ4. What vaccine did you receive?	1.Comirnaty (Biontech/Pfizer)<br>2.COVID-19 Vaccine Moderna (Moderna)<br>4.Vaxzevria (AstraZeneca)<br>5. COVID-19 Vaccine Jansen (Johnson & Johnson)<br>3.Don't know

S2 Variables

For both MuSPAD and COVIMOD, the number of contacts was truncated at 100. In COVIMOD, the truncation occurred for group contacts per location, rather than the total number of contacts. Since MuSPAD collected aggregated contact data by person categories rather than location, only the number of non-household contacts and the total number of contacts was truncated at 100 for MuSPAD.

In both studies, age was included as a continuous variable in the analyses and pre-existence of comorbidities was recorded as a binary variable. The gender category “diverse” was excluded from inferential analyses due to few observations.

In general, similar variables were considered for both MuSPAD and COVIMOD; a detailed overview of all variables can be found in the following table. Housing situation was operationalised in the form of household groups of different sizes (MuSPAD and COVIMOD) and the average number of square meters available to each household member (MuSPAD only). Further variables assessed the average income and occupational situation of participants, information on the infection status of household members, and the number of minor children living in the household.

Some analogous variables were defined slightly differently in MuSPAD and COVIMOD. In addition to average income, socioeconomic status for MuSPAD included the highest acquired level of education, whereas COVIMOD used occupational rank. To account for changing containment strategies, for analyses in MuSPAD we used the variable stringency index from the Oxford COVID-19 Government Response Tracker (OxCGR) with the numeric score grouped into categories [25]; in analyses in COVIMOD, the survey wave was used as a proxy for changing conditions and regulations over time.

Additional variables were included according data availability. Home office activity and smoking status were considered for MuSPAD. To account for the reported incidence as orientation for an individual’s behaviour, 7-day incidences per federal state, as provided by

the Robert Koch-Institute (RKI), were grouped into a categorical covariate for the MuSPAD analysis [26]. Since COVIMOD was partially longitudinal, the number of completed survey waves was considered to account for survey fatigue. COVIMOD also included information on whether an individual had been in quarantine or isolation in the past 7 days.

Table S2.0.1 Definitions of variables for MuSPAD and COVIMOD.

Variable	MuSPAD variable levels	COVIMOD variable levels
Household contacts	*Integer*<br>*Note: categorised into "no household contact", "1 household contact", "2 household contacts", and "3 or more household contacts" for seropositivity logistic regression models*	*Integer*
Non-household contacts	*Integer*<br>*Note: categorised into "no non-household contact", "1-3 non-household contacts", "4-10 non-household contacts", and "11 or more non-household contacts" for seropositivity logistic regression models*	*Integer*
Total contacts	*Integer*<br>*Note: categorised into "up to 3 contacts", "4-10 contacts", and "11 or more contacts" for seropositivity logistic regression models*	*Integer*
Age	*Integer*	*Integer*
Vaccination status	No vaccination reported<br>Only one vaccine dose reported<br>Complete vaccination reported<br>Unknown vaccination status	Not Vaccinated<br>Partially Vaccinated<br>Fully Vaccinated
Immunity status	No vaccination and no knowledge of infection<br>Vaccination but no knowledge of infection<br>No vaccination but knowledge of infection<br>Vaccination and knowledge of infection	No vaccination and no knowledge of infection<br>Vaccination but no knowledge of infection<br>No vaccination but knowledge of infection<br>Vaccination and knowledge of infection
Contact with infected individual	No<br>Yes<br>Unclear contact	No<br>Yes<br>Unclear contact
Child living in household	No child living in household<br>Youngest child aged up to 5 years<br>Youngest child aged 6 to 10 years<br>Youngest child aged 11 to 17 years<br>Living with child of unknown age	No child living in household<br>Youngest child aged up to 4 years<br>Youngest child aged 5 to 9 years<br>Youngest child aged 10 to 14 years<br>Youngest child aged 15 to 19 years
Employment	Other work sector<br>Health and Education<br>Retired<br>Unemployed	Other work sector<br>Health and Education<br>Retired<br>Unemployed
Working from home	No work in homeoffice reported<br>Work at least partly in homeoffice<br>Retired<br>Unemployed	N/A
COVID test status of household members	No household member was tested<br>Negative test result of household member<br>Positive test result of household member<br>Unclear household testing	No household member was tested<br>Negative test result of household member<br>Positive test result of household member<br>Unclear household testing
Pre-existing condition	No pre-existing condition<br>Pre-existing condition<br>Unknown or not reported	No pre-existing condition<br>Pre-existing condition<br>Unknown or not reported
Socioeconomic status	Academic education / University degree<br>Apprenticeship or Professional school diploma<br>Qualification for university entrance<br>9 or 10 years of schooling<br>No professional qualification or still in training	1. Managerial<br>2. Clerical<br>3. Manual<br>4. Self employed<br>5. Retired / Unemployed

**Variable**	**MuSPAD variable levels**	**COVIMOD variable levels**
Monthly household income	less than 2000 Euro<br>2000 to less than 6000 Euro<br>6000 Euro or more	less than 2001 Euro<br>2001 to 5000 Euro<br>More than 5000 Euro<br>Unknown income
Gender	Female<br>Male<br>Divers	Female<br>Male<br>Diverse
Vaccination proportion (proportion of the German population that is vaccinated)	*Number between 0 and 1*	*Number between 0 and 1*
Location	Aachen 2<br>Chemnitz 1<br>Osnabrück 2<br>Magdeburg 2<br>Greifswald<br>Hanover<br>Chemnitz 2<br>*Note: since the timing of the surveys differed in each location, this also serves as a proxy for time for MuSPAD*	Baden-Württemberg<br>Bayern<br>Berlin<br>Brandenburg<br>Bremen<br>Hamburg<br>Hessen<br>Mecklenburg-Vorpommern<br>Niedersachsen<br>Nordrhein-Westfalen<br>Rheinland-Pfalz<br>Saarland<br>Sachse<br>Sachsen-Anhalt<br>Schleswig-Holstein<br>Thüringen<br>*Note: COVIMOD has data on federal states since it is nation-wide rather than associated with specific study centers*
Household size	Living alone<br>2-person household<br>3-person household<br>4-person household<br>Living with more than 4 people	Living alone<br>2-person household<br>3-person household<br>4-person household<br>Living with more than 4 people
Household density	up to 30 sqm/person<br>more than 30 to 40 sqm/person<br>more than 40 to 50 sqm/person<br>more than 50 to 60 sqm/person<br>more than 60 to 70 sqm/person<br>more than 70 sqm/person	N/A
Number of survey waves completed by participant up to and including this wave	N/A	*Integer (range 1-30, median 6, mean 8.7)*
Isolation or quarantine due to COVID-19	N/A	In quarantine/isolation last 7 days<br>No quarantine/isolation last 7 days<br>Unknown quarantine/isolation
Survey wave number	N/A<br>*Note: Location (Standort) also differs by time*	*Integer (range 18-33)*
7-day-incidence	up to 50<br>between 50 and 100<br>over 100	N/A
Stringency index	up to 70<br>up to 80<br>over 80	N/A
Smoking behaviour	Never smoked<br>Ex-smoker<br>Smoker, occasionally or daily<br>Invalid or missing response	N/A

S2.1 DAGs and adjustment sets

The selection strategy of potential covariates was based on subject-matter knowledge and expert judgement. DAGs were initially based on data available for the MuSPAD study but were then also used for the COVIMOD study. DAGs were build with the help of dagitty.net [27]. The exposure is indicated with "►", the outcome with "I". The colouring convention designates exposures and their ancestors in green, outcomes and their ancestors in blue, and confounding covariates in red. Variables not directly observed are represented within an ellipse.

Figure S2.1.1 DAG for the impact of assumed immunity status on household contacts.

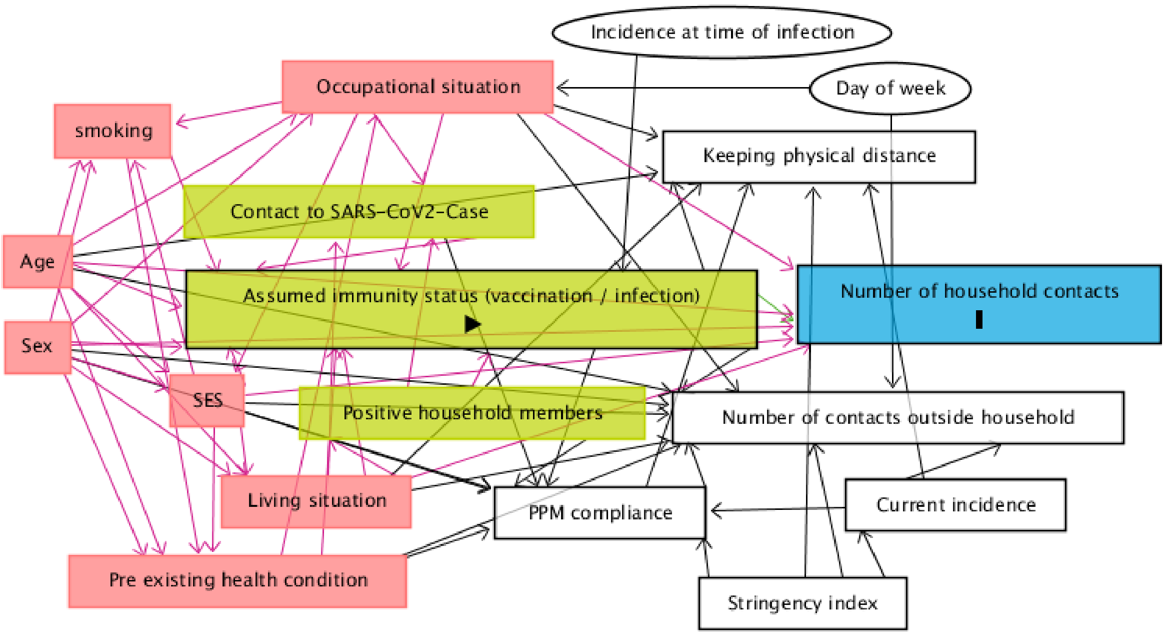

Figure S2.1.2 DAG for the impact of assumed immunity status on non-household contacts.

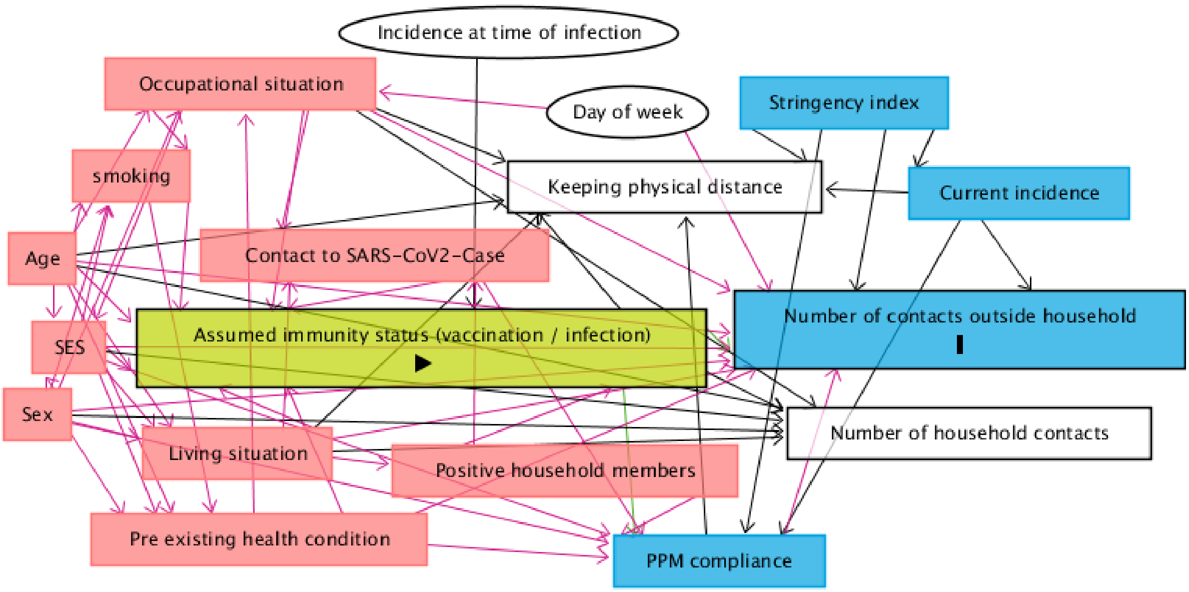

Figure S2.1.3 DAG for the impact of household contacts on seropositivity due to infection.

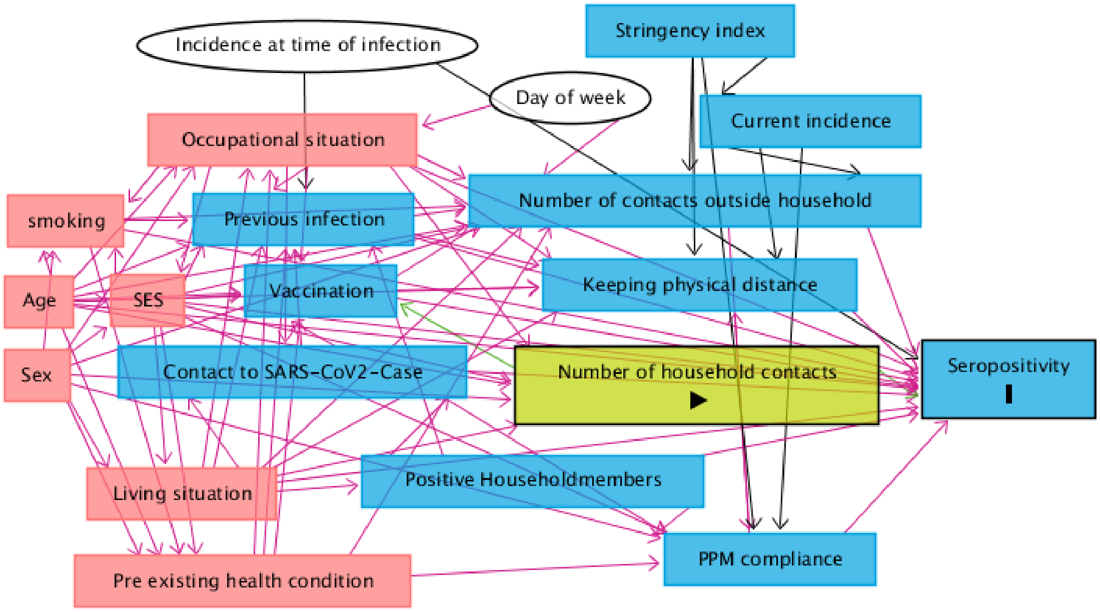

Figure S2.1.4 DAG for the impact of non-household contacts on seropositivity due to infection.

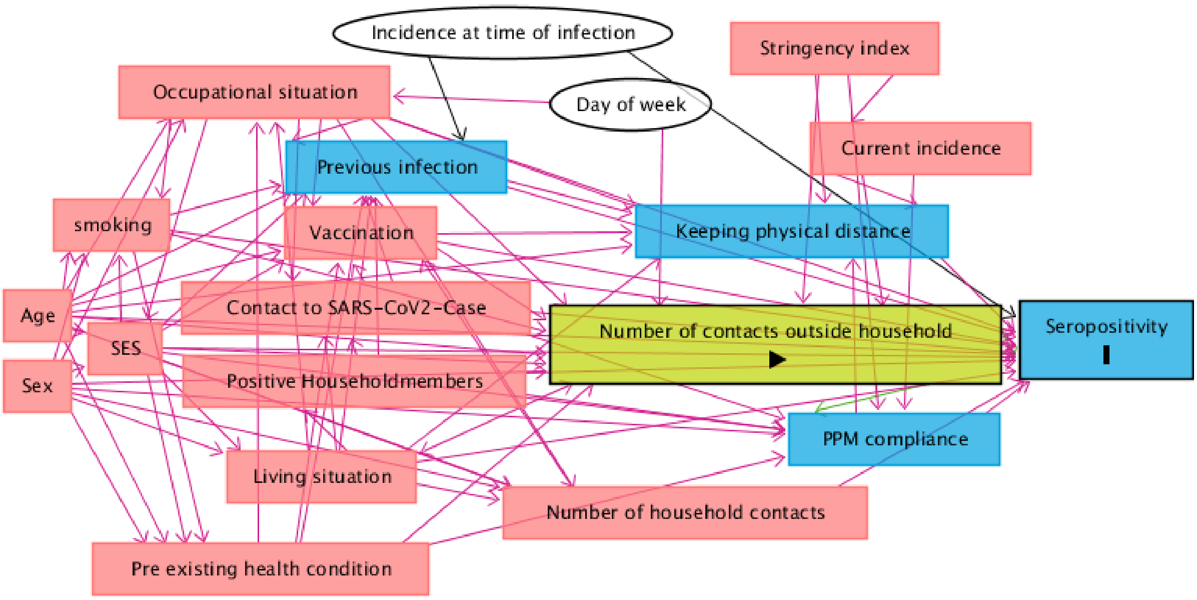

Figure S2.1.5 DAG for the impact of living with a child in the household on seropositivity due to infection.

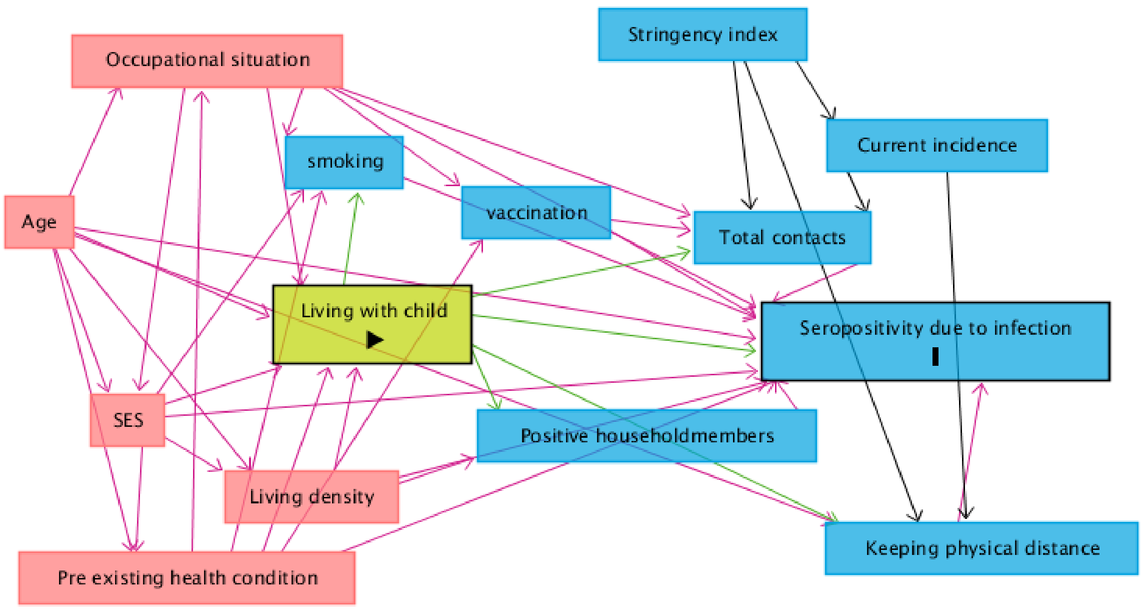

Table S2.1.1 Adjusted variables in regression models for MuSPAD and COVIMOD.
List of which variables were used for each model given the restriction of different variables.

Model	MuSPAD adjusted variables	COVIMOD adjusted variables
Negative binomial regression models for total contacts	Age,<br>Gender,<br>Location (random effects),<br>Employment,<br>Working from home,<br>Monthly household income,<br>Socioeconomic status,<br>Child living in household,<br>Contact with infected individual,<br>COVID test status of household members,<br>Pre-existing condition,<br>Vaccination proportion	Age,<br>Gender,<br>Location (random effects),<br>Employment,<br>Monthly household income,<br>Socioeconomic status,<br>Child living in household,<br>Number of completed waves,<br>Survey wave (random effects),<br>Quarantine or isolation,<br>Contact with infected individual,<br>COVID test status of household members,<br>Pre-existing condition,<br>Vaccination proportion
Negative binomial regression models for household contacts	Age,<br>Gender,<br>Location (random effects),<br>Employment,<br>Working from home,<br>Monthly household income,<br>Socioeconomic status,<br>Child living in household,<br>Vaccination proportion	Age,<br>Gender,<br>Location,<br>Employment,<br>Monthly household income,<br>Socioeconomic status,<br>Child living in household,<br>Number of completed waves,<br>Survey wave (random effects),<br>Quarantine or isolation,<br>Vaccination proportion
Negative binomial regression models for non-household contacts	Age,<br>Gender,<br>Location (random effects),<br>Employment,<br>Working from home,<br>Monthly household income,<br>Socioeconomic status,<br>Child living in household,<br>Contact with infected individual,<br>COVID test status of household members,<br>Pre-existing condition,<br>Household size,<br>Vaccination proportion	Age,<br>Gender,<br>Location,<br>Employment,<br>Monthly household income,<br>Socioeconomic status,<br>Child living in household,<br>Number of completed waves,<br>Survey wave (random effects),<br>Quarantine or isolation,<br>Contact with infected individual,<br>COVID test status of household members,<br>Pre-existing condition,<br>Household size,<br>Vaccination proportion

Model	MuSPAD adjusted variables	COVIMOD adjusted variables
Logistic regression models for seropositivity due to infection for total contacts	Age,<br>Gender,<br>Location (random effects),<br>Employment,<br>Working from home,<br>Monthly household income,<br>Socioeconomic status,<br>Child living in household,<br>Pre-existing condition,<br>7-day incidence,<br>Stringency index,<br>Vaccination status,<br>Smoking,<br>Vaccination proportion	N/A
Logistic regression models for seropositivity due to infection for household contacts	Age,<br>Gender,<br>Location (random effects),<br>Employment,<br>Working from home,<br>Monthly household income,<br>Socioeconomic status,<br>Child living in household,<br>Vaccination proportion	N/A
Logistic regression models for seropositivity due to infection for non-household contacts	Age,<br>Gender,<br>Location (random effects),<br>Employment,<br>Working from home,<br>Monthly household income,<br>Socioeconomic status,<br>Child living in household,<br>Pre-existing condition,<br>Household size,<br>7-day-incidence,<br>Stringency index,<br>Vaccination status,<br>Smoking,<br>Vaccination proportion	N/A

S3 Demographics

S3.1 Study population

Figure S3.1.1 Flow chart of the MuSPAD study population.
The total population of the MuSPAD study and the subsamples of the presented analyses are displayed. 52,65% of all study participants were included in 2021 after the start of the vaccination campaign. The questionnaire including contact behaviour was only offered to 2021 participants on a voluntary basis. A response rate of 60,24% resulted in a sample size of 12.641 for the contact evaluations, that were conducted in the total study sample as well as gender subgroups. Sample size varied in each analysis due to different adjustment sets for the different contact settings (total contacts, contacts in and contacts outside household).

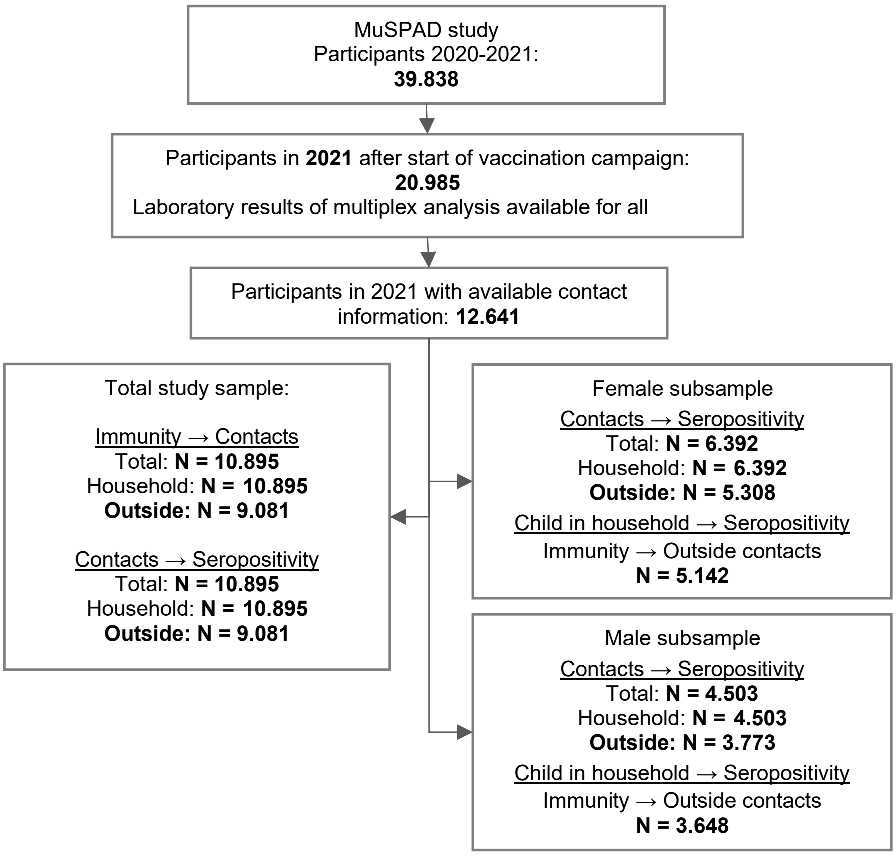

Figure S3.1.2 Inclusion and exclusion of COVIMOD survey entries.
An entry is defined here as one survey wave of data for one participant; if a participant completed numerous waves of the survey, then they have one entry for each completed survey wave.

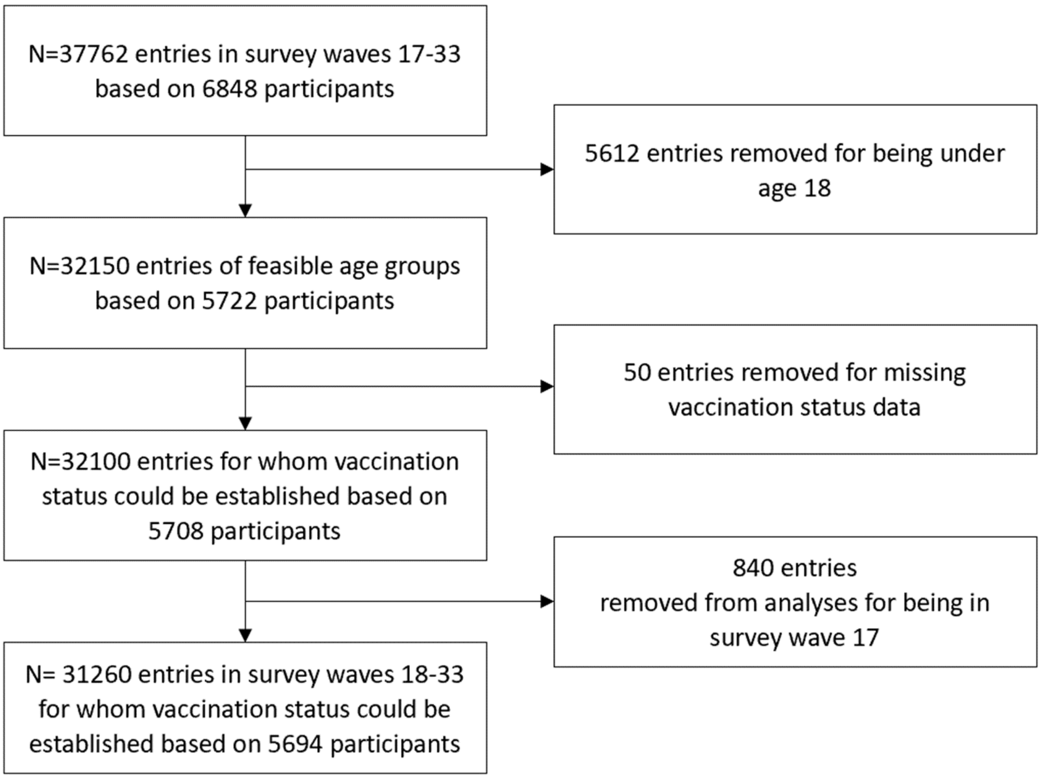

Table S3.1.1 Demographic characteristics of the MuSPAD and COVIMOD studies. Absolute and relative frequencies are given for categorical variables, minimum (min), maximum (max), mean (SD) and median (IQR) for continuous variables.

Attribute	MuSPAD		COVIMOD	
	N	%	N	%
Sample size: responses (participants)	12641 (12641)	100.00%	31260 (5694)	100.00%
Analysis period	27 January to 17 August 2021		24 February to 31 December 2021	
Study centres	Aachen, Osnabrück, Greifswald, Chemnitz, Magdeburg, Hanover		Germany-wide	
**Location**				
Aachen	1785	14.1%	-	-
Chemnitz 1	1921	15.2%	-	-
Osnabrück	2128	16.8%	-	-
Magdeburg	1840	14.6%	-	-
Greifswald	1693	13.4%	-	-
Hanover	2309	18.3%	-	-
Chemnitz 2	965	7.6%	-	-
Baden-Württemberg	-	-	3851	12.3%
Bavaria	-	-	4906	15.7%
Berlin	-	-	1531	4.9%
Brandenburg	-	-	858	2.7%
Bremen	-	-	250	0.8%
Hamburg	-	-	924	3.0%
Hesse	-	-	2535	8.1%
Mecklenburg-Vorpommern	-	-	546	1.7%
Lower Saxony	-	-	2629	8.4%
North Rhine-Westphalia	-	-	6961	22.3%
Rhineland-Palatinate	-	-	1471	4.7%
Saarland	-	-	324	1.0%
Saxony	-	-	1003	3.2%
Saxony-Anhalt	-	-	878	2.8%
Schleswig-Holstein	-	-	1384	4.4%
Thuringia	-	-	1209	3.9%
**Sex**				
Female	7449	58.9%	15016	48.0%
Male	5189	41.1%	16177	51.7%
Diverse	<6	-	59	16.3%
Missing	0	0.0%	8	<0.1%
**Age**				
Min | Max	18 | 99		18 | 92	
Median (IQR)	55 (42 - 66)		55 (40 - 66)	
Mean (SD)	54.0 (16.0)		52.8 (16.3)	
Missing	38		261	
**Vaccination status**				
No vaccination reported	7549	59.7%	9447	30.2%
Only one vaccine dose reported	2351	18.6%	4726	15.1%
Complete vaccination reported[1]	2691	21.3%	17087	54.7%
Unknown vaccination status	50	0.4%	0	0.0%
**Previous SARS-CoV-2 tests**				
No test performed	5019	39.7%	17001	54.4%
All performed SARS-CoV-2 tests were negative	6984	55.3%	12573	40.2%
At least one positive SARS-CoV-2 test reported	590	4.7%	1251	4.0%
Invalid or missing response	48	0.4%	435	1.4%
**Multiplex results in MuSPAD[2]**				
No SARS-CoV- 2 antibodies	7568	59.9%	-	-
SARS-CoV-2 anti-S, anti-RBD	4426	35.0%	-	-
SARS-CoV-2 anti-NC, anti-S, anti-RBD	647	5.1%	-	-
**Immunity status[3]**				
No vaccination and no knowledge of infection	7112	56.3%	9100	29.1%
Vaccination but no knowledge of infection	4939	39.1%	20909	66.9%
No vaccination but knowledge of infection	487	3.9%	347	1.1%
Vaccination and knowledge of infection	103	0.8%	904	2.9%
**Serostatus**				
No vaccination and no previous infection	7499	59.3%	-	-
Vaccination but no previous infection	4131	32.7%	-	-
No vaccination but previous infection	831	6.6%	-	-
Vaccination and previous infection	180	1.4%	-	-
**Previous infection**				
no previous infection	11630	92.0%	-	-
previous infection	1011	8.0%	-	-
**Tests of household members**				
No household member was tested	3076	24.3%	13295	42.5%
Negative test result of household member	4697	37.2%	5935	19.0%
Positive test result of household member	461	3.7%	187	0.6%
Unclear household testing	4407	34.8%	11843	37.9%
**Housing situation**				
Living alone	2056	16.3%	11359	36.3%
2-person household	5163	40.8%	13536	43.3%
3-person household	1505	11.9%	4306	13.8%
4-person household	1278	10.1%	1545	4.9%
More than 4 people in household	370	2.9%	514	1.6%

Attribute	MuSPAD		COVIMOD	
	N	%	N	%
Missing	2269	17.9%	-	-
**Housing density**				
Up to 30 sqm / person	1890	15.0%	-	-
More than 30 to 40 sqm / person	2280	18.0%	-	-
More than 40 to 50 sqm / person	1929	15.3%	-	-
More than 50 to 60 sqm / person	1491	11.8%	-	-
More than 60 to 70 sqm / person	917	7.3%	-	-
More than 70 sqm / person	1510	11.9%	-	-
Missing	2624	20.8%	-	-
**Child in household**				
No child living in household	9213	72.9%	29752	95.2%
Child living in household	3428	27.1%	1508	4.8%
Age of youngest child				
up to 5 years	763	6.0%	-	-
6 to 10 years	536	4.2%	-	-
11 to 17 years	888	7.0%	-	-
Missing	1241	9.8%	-	-
up to 4 years	-	-	244	0.8%
5 to 9 years	-	-	179	0.6%
10 to 14 years	-	-	348	1.1%
15 to 19 years	-	-	737	2.4%
**Employment status**				
Retired	3752	29.7%	9015	28.8%
Health and Education	2160	17.1%	326	1.0%
Unemployed	1283	10.2%	2024	6.5%
Other work sector	4728	37.4%	19895	63.6%
Missing	718	5.7%	-	-
**Work in home office**				
No work in home office reported	4799	38.0%	-	-
Work at least partly in home office	2807	22.2%	-	-
Retired	3752	29.7%	-	-
Unemployed	1283	10.2%	-	-
**Monthly net household income**				
less than 2000 Euro	4172	33.0%	-	-
2000 to less than 6000 Euro	6214	49.2%	-	-
6000 Euro or more	757	6.0%	-	-
Less than 2001 Euro	-	-	4665	14.9%
2001 to 5000 Euro	-	-	5474	17.5%
More 5000 Euro	-	-	815	2.6%
Missing	1498	11.9%	20306	65.0%
**Educational qualification**				
Academic education / University degree	4822	38.2%		-
Apprenticeship or Professional school diploma	2704	21.4%	-	-
Qualification for university entrance	1266	10.0%	-	-
9 or 10 years of schooling	2828	22.4%	-	-
No professional qualification or still in training	93	0.7%	-	-
Missing	928	7.3%	-	-
**Socioeconomic status**				
1. Managerial	-	-	6824	21.8%
2. Clerical	-	-	9580	30.6%
3. Manual	-	-	1507	4.8%
4. Self employed	-	-	1296	4.1%
5. Retired / Unemployed	-	-	12053	38.6%
**Contact to COVID-19 cases**				
No	8112	64.2%	27814	89.0%
Yes	1566	12.4%	925	3.0%
Unclear contact	2963	23.4%	2521	8.1%
**Pre-existing health conditions**				
No pre-existing condition	7925	62.7%	16887	54.0%
Pre-existing condition	4644	36.7%	11447	36.6%
Thereof				
Hypertension	3346	50.0%	-	-
Diabetes	637	9.5%	-	-
Cardiovascular disease	1043	15.6%	-	-
Chronic pulmonary disease	870	13.0%	-	-
Present immunodeficiency	490	7.3%	-	-
Cancer	300	4.5%	-	-
Unknown or not reported	72	0.6%	2926	9.4%
**Smoking status**				
Never smoked	7083	56.0%	-	-
Ex-smoker	3611	28.6%	-	-
Smoker, occasionally or daily	1903	15.1%	-	-
Invalid or missing response	44	0.3%	-	-
**Quarantine or isolation**				
In quarantine/isolation last 7 days	-	-	534	1.7%
No quarantine/isolation last 7 days	-	-	30480	97.5%
Unknown quarantine/isolation	-	-	246	0.8%
**Survey wave**				
24.02.-03.03. (W18)	-	-	1263	4.0%
17.03.-26.03. (W19)	-	-	1263	4.0%
07.04.-15.04. (W20)	-	-	1268	4.1%
12.05.-24.05. (W21)	-	-	2091	6.7%

Attribute	MuSPAD		COVIMOD	
	N	%	N	%
26.05.-03.06. (W22)	-	-	2089	6.7%
09.06.-22.06. (W23)	-	-	2127	6.8%
07.07.-19.07. (W24)	-	-	2123	6.8%
04.08.-13.08. (W25)	-	-	2152	6.9%
01.09.-14.09. (W26)	-	-	2142	6.9%
22.09.-06.10. (W27)	-	-	2126	6.8%
08.10.-20.10. (W28)	-	-	2125	6.8%
22.10.-02.11. (W29)	-	-	2083	6.7%
03.11.-09.11. (W30)	-	-	2077	6.6%
17.11.-23.11. (W31)	-	-	2112	6.8%
08.12.-17.12. (W32)	-	-	2109	6.7%
24.12.-31.12. (W33)	-	-	2110	6.7%
**Number of survey waves completed**				
Min | Max		-	1 | 30	
Median (IQR)		-	6 (3 - 14)	
Mean (SD)		-	8.7 (6.9)	

[1] Single dose of Johnson&Johnson was considered as completely vaccinated
[2] Antibodies against spike, receptor-binding domain or nucleocapsid protein
[3] Based on self-reported vaccinations and infections

S3.2 Restricted timeframe

Table S3.2.1 Summary characteristics of the MuSPAD and COVIMOD samples for restricted collection period form 24 February to 17 August, 2021.
Absolute and relative frequencies are given for categorical variables, minimum (min), maximum (Max), mean (SD) and median (IQR) for continuous variables.

Attribute	MuSPAD	COVIMOD
Sample size: responses (participants)	10856 (10856)	14376 (3371)
Analysis period	24 February to 17 August 2021	24 February to 17 August 2021
Study centres	Osnabrück, Greifswald, Chemnitz, Magdeburg, Hanover	Germany-wide
**Sex**		
Female	6434 (59.3%)	6841 (47.7%)
Male	4420 (40.7%)	7505 (52.3%)
Diverse	<6 (0.0%)	26 (0.2%)
Missing	0 (0.0%)	4 (<0.1%)
**Age**		
Mean (SD)	54.5 (15.8)	51.8 (16.6)
Median (IQR)	56.0 (44.0 - 67.0)	54 (38-66)
Min | Max	18.0 | 99.0	18 | 92
Missing	26	145
**Vaccination status**		
No vaccination reported	5816 (53.6%)	7278 (50.6%)
Only one vaccine dose reported	2320 (21.4%)	3418 (23.8%)
Complete vaccination reported[1]	2676 (24.6%)	3680 (25.6%)
Unknown vaccination status	44 (0.4%)	0 (0.0%)
**Previous SARS-CoV-2 tests**		
No test performed	4048 (37.3%)	7938 (55.2%)
All performed SARS-CoV-2 tests were negative	6242 (57.5%)	5670 (39.4%)
At least one positive SARS-CoV-2 test reported	524 (4.8%)	546 (3.8%)
Invalid or missing response	42 (0.4%)	222 (1.5%)
**Multiplex results in MuSPAD[2]**		
No SARS-CoV- 2 antibodies	5898 (54.3%)	-
SARS-CoV-2 anti-S, anti-RBD	4362 (40.2%)	-
SARS-CoV-2 anti-NC, anti-S, anti-RBD	596 (5.5%)	-
**Immunity status[3]**		
No vaccination and no knowledge of infection	5437 (50.1%)	7044 (49.0%)
Vaccination but no knowledge of infection	4895 (45.1%)	6786 (47.2%)
No vaccination but knowledge of infection	423 (3.9%)	234 (1.6%)
Vaccination and knowledge of infection	101 (0.9%)	312 (2.2%)
**Total contacts**		
Reported contacts in study population	103913	35929
Mean (SD) per person in 24h	9.6 (12.8)	2.5 (7.9)
Median (IQR) per person in 24h	6.0 (3.0 - 12.0)	1.0 (0.0 - 2.0)
Min | Max	0.0 | 100.0	0.0 | 302.0
**Household contacts**		
Reported contacts in study population	19232	11286
Mean (SD) per person in 24h	1.8 (1.2)	0.8 (0.9)
Median (IQR) per person in 24h	2.0 (1.0 - 2.0)	1.0 (0.0 – 1.0)
Min | Max	0.0 | 8.0	0.0 | 9.0
**Non-household contacts**		
Reported contacts in study population	84780	24643
Mean (SD) per person in 24h	7.8 (12.7)	1.7 (7.8)
Median (IQR) per person in 24h	4.0 (1.0 - 10.0)	0.0 (0.0 – 1.0)
Min | Max	0.0 | 100.0	0.0 | 300.0

[1] Single dose of Johnson&Johnson was considered as completely vaccinated
[2] Antibodies against spike, receptor-binding domain or nucleocapsid protein
[3] Based on self-reported vaccinations and infections

S3.3 Contact groups in MuSPAD

Table S3.3.1 Contact groups of the MuSPAD sample.
Absolute and relative frequencies are given for categorical variables.

Attribute	Category	MuSPAD
All contacts		
	no contacts	192 (1.5%)
	1-3 contacts	4084 (32.3%)
	4-10 contacts	4764 (37.7%)
	11 or more contacts	3601 (28.5%)
Household contacts		
	no inside contact	1247 (9.9%)
	1 inside contact	5008 (39.6%)
	2 inside contacts	3367 (26.6%)
	3 or more inside contacts	3019 (23.9%)
Non-household contacts		
	no contacts outside household	2597 (20.5%)
	1-3 contacts outside household	3726 (29.5%)
	4-10 contacts outside household	3322 (26.3%)
	11 or more contacts outside household	2996 (23.7%)

S4 Contact behaviour over time

Figure S4.0.1 Timing of MuSPAD collection periods by study centre.
Top: Timeline of recruitment at each study site and the corresponding stringency index to quantify national containment measures at the respective time points; the marked background areas highlight periods with distinct legal restrictions in terms of contact numbers implemented by the German government. Bottom: Transmission activity in form of nationwide as well as regional incidence rates in the respective federal states where selected study sites are located, coded by tone of colour.

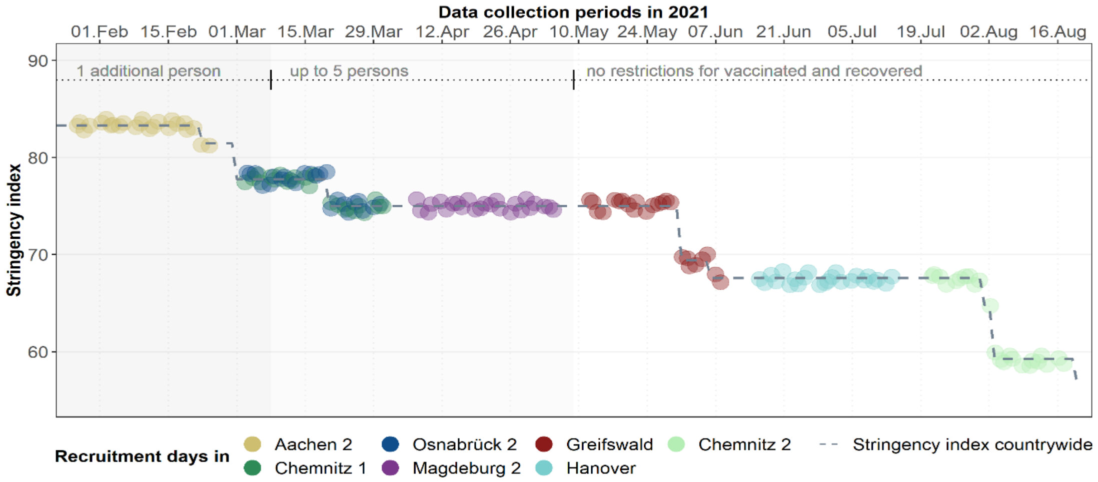

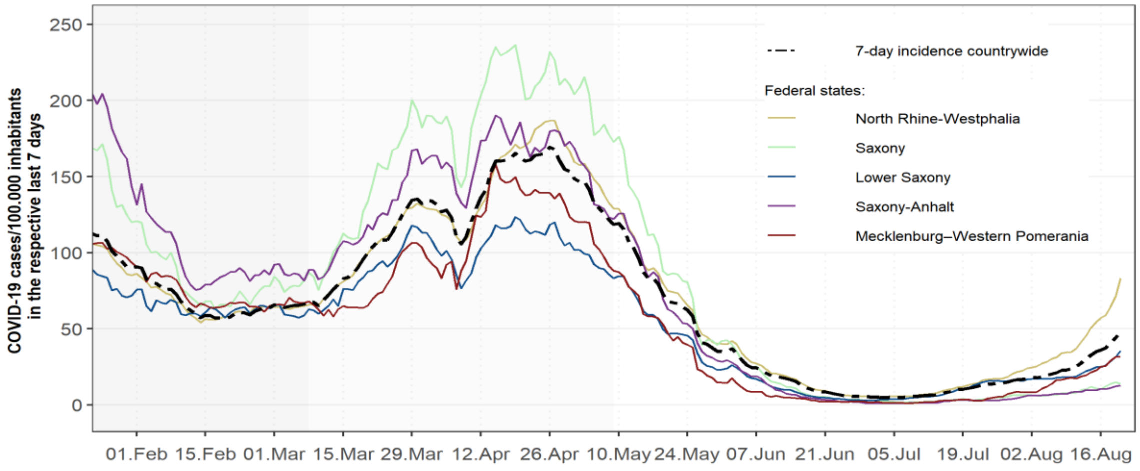

S4.1 Boxplots

We created boxplots to show contact numbers for each contact setting over strata of immunity, vaccination, and serostatus over two (MuSPAD) or three (COVIMOD) phases (arranged in rows) based on restrictions in place during each period. To better illustrate low contact numbers of the right skewed count variables, the y-axis was transformed to log-scale. For COVIMOD, the y-axis was limited to 100 but outliers were still considered in the calculation of the boxplots and mean.

Figure S4.1.1 Contact numbers stratified by immunity status.
Boxplot of contact numbers of all, household, and non-household contacts over different phases of the pandemic stratified by status of immunity reported by (a) MuSPAD participants and (b) COVIMOD participants. Boxplots display the median and white dots show the mean contacts per type and stratum. To better illustrate low contact numbers of the right skewed count variables, the y-axis was transformed to log-scale.

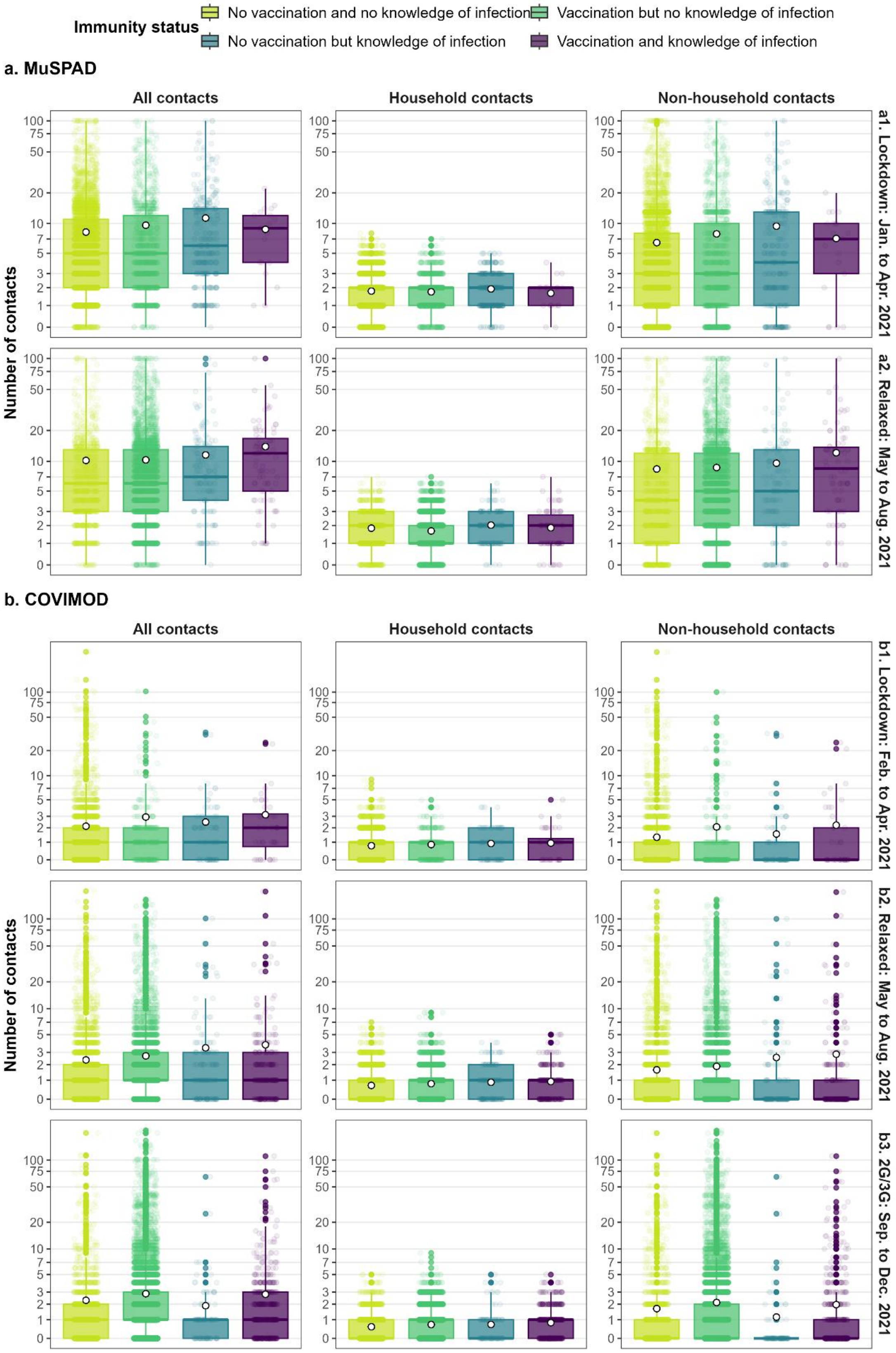

Figure S4.1.2 Contact numbers stratified by vaccination status.
Boxplot of contact numbers of all, household, and non-household contacts over different phases of the pandemic stratified by vaccination status of (a) MuSPAD participants and (b) COVIMOD participants. Boxplots display the median and white dots show the mean contacts per type and stratum. To better illustrate low contact numbers of the right skewed count variables, the y-axis was transformed to log-scale.

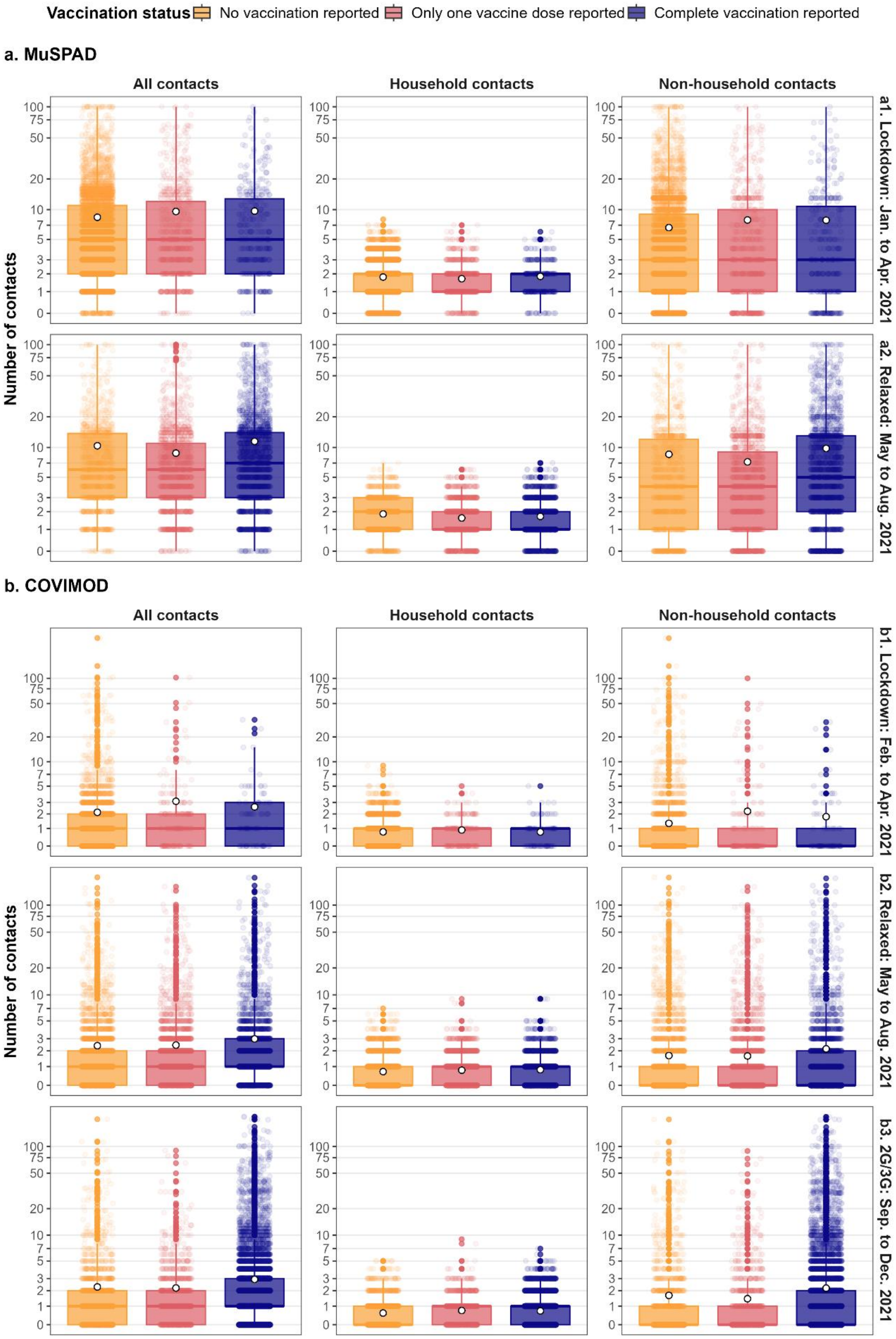

Figure S4.1.3 Contact numbers stratified by serostatus.
Boxplot of contact numbers of all, household, and non-household contacts over different phases of the pandemic stratified by serostatus of MuSPAD participants. Boxplots display the median and white dots show the mean contacts per type and stratum. To better illustrate low contact numbers of the right skewed count variables, the y-axis was transformed to log-scale.

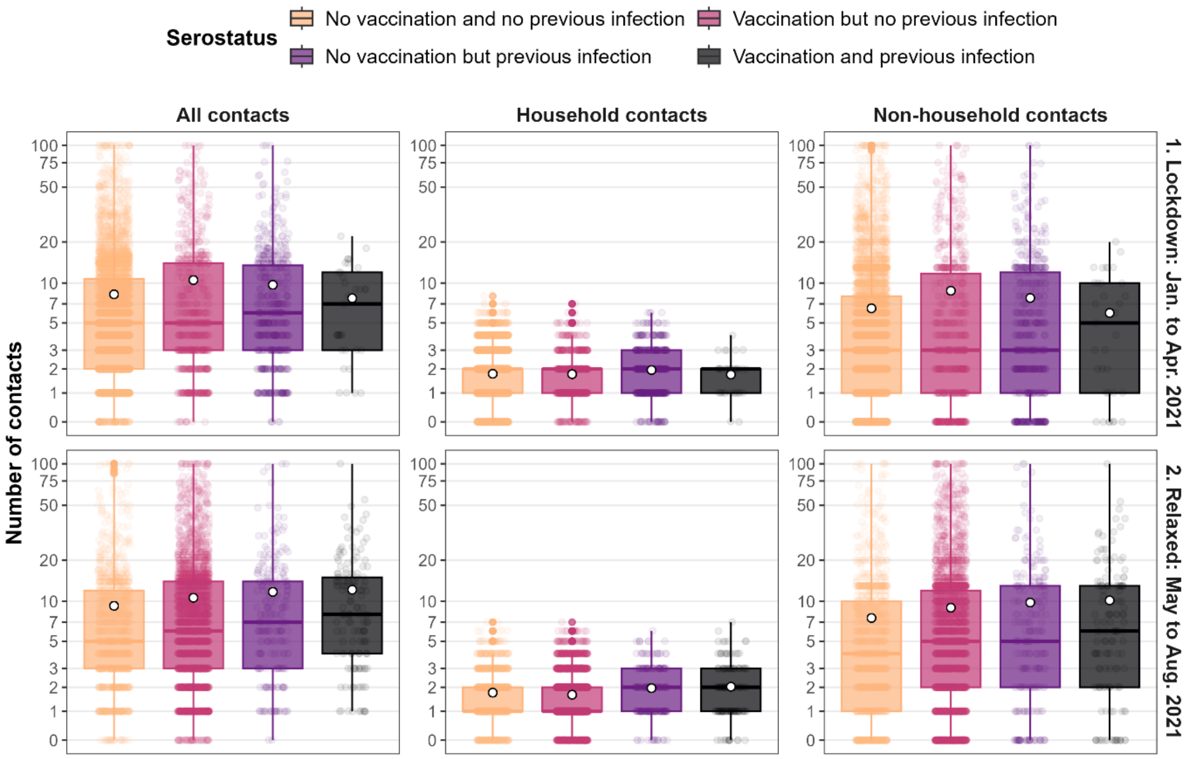

S4.2 Mean Contact Plots

Figure S4.2.1 Mean number of contacts over time stratified by study, immunity status, vaccination status, and serostatus.

The plots illustrate the mean number of overall contacts for a) MuSPAD stratified by immunity status, b) COVIMOD stratified by immunity status, c) MuSPAD stratified by vaccination status, d) COVIMOD stratified by vaccination status, e) MuSPAD stratified by serostatus, based on laboratory results allowing the distinction between infection- or vaccine-acquired antibodies according to the detection of anti-NC, corrected by self-reported vaccination and test history. The dashed grey line represents the stringency of government restrictions in place each day. Points mark measured means; smoothing to indicate temporal trends was done with the method “loess”. The shaded regions indicate the 95% confidence interval of the estimates of the means derived with the loess smoothing method. For MuSPAD, contact behaviour was aggregated as weeks to account for varying contact behaviour on different days of the working week and weekend; for COVIMOD, the mean number of contacts was calculated by survey wave. Note differences in the y-axes between plots.

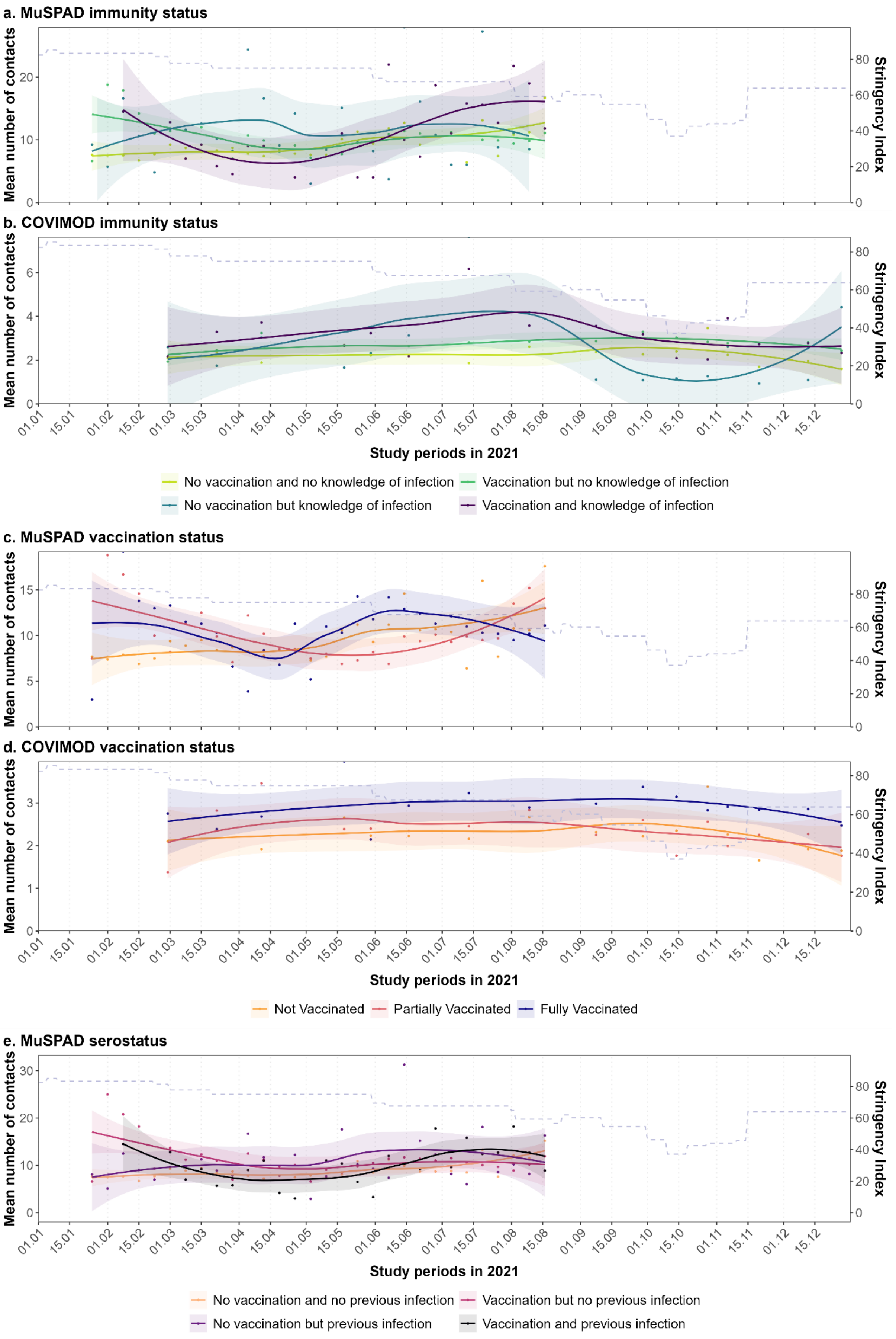
a. MuSPAD immunity status
Mean number of contacts
Stringency Index
b. COVIMOD immunity status
Mean number of contacts
Stringency Index
01.01
15.01
01.02
15.02
01.03
15.03
01.04
15.04
01.05
15.05
01.06
15.06
01.07
15.07
01.08
15.08
01.09
15.09
01.10
15.10
01.11
15.11
01.12
15.12
Study periods in 2021
No vaccination and no knowledge of infection
Vaccination but no knowledge of infection
No vaccination but knowledge of infection
Vaccination and knowledge of infection
c. MuSPAD vaccination status
Mean number of contacts
Stringency Index
d. COVIMOD vaccination status
Mean number of contacts
Stringency Index
Study periods in 2021
Not Vaccinated
Partially Vaccinated
Fully Vaccinated
e. MuSPAD serostatus
Mean number of contacts
Stringency Index
Study periods in 2021
No vaccination and no previous infection
Vaccination but no previous infection
No vaccination but previous infection
Vaccination and previous infection

S5 Sex-specific subgroup analyses (MuSPAD only)

S5.1 Summary characteristics by female and male subsamples

Table S5.1.1 Summary demographics of the study population, stratified by females and males. Sociodemographic and health characteristics for the total study sample as well as stratified by the two gender groups. Descriptive statistics present absolute and relative frequencies.

Attribute	Female subsample		Male subsample	
	N	%	N	%
Total	7449	100.0%	5189	100.0%
**Location**				
Aachen 2	1015	13.6%	769	14.8%
Chemnitz 1	1069	14.4%	852	16.4%
Osnabrück 2	1277	17.1%	851	16.4%
Magdeburg 2	1137	15.3%	702	13.5%
Greifswald	1078	14.5%	615	11.9%
Hanover	1273	17.1%	1035	19.9%
Chemnitz 2	600	8.1%	365	7.0%
**Age**				
Min | Max	18.0 | 99.0		18.0 | 95.0	
Median (IQR)	55.0 (42.0 - 65.0)		56.0 (44.0 - 68.0)	
Mean (SD)	53.2 (15.9)		54.9 (16.2)	
Missing	23		15	
**Vaccination status**				
No vaccination reported	4379	58.8%	3168	61.1%
Only one vaccine dose reported	1391	18.7%	960	18.5%
Complete vaccination reported[1]	1650	22.2%	1040	20.0%
Unknown vaccination status	29	0.4%	21	0.4%
**Previous SARS-CoV2 tests**				
No test performed	2778	37.3%	2239	43.1%
All performed SARS-CoV-2 tests were negative	4292	57.6%	2692	51.9%
At least one positive SARS-CoV-2 test reported	351	4.7%	238	4.6%
Invalid or missing response	28	0.4%	20	0.4%
**Multiplex results in MuSPAD[2]**				
no antibodies	4378	58.8%	3189	61.5%
anti-S, anti-Rb	2692	36.1%	1733	33.4%
anti-Nc, anti-S, anti-Rb	379	5.1%	267	5.1%
**Immunity status[3]**				
No vaccination and no knowledge of infection reported	4129	55.4%	2982	57.5%
Vaccination but no knowledge of infection reported	2969	39.9%	1969	37.9%
No vaccination but knowledge of infection reported	279	3.7%	207	4.0%
Vaccination and knowledge of infection reported	72	1.0%	31	0.6%
**Child in household**				
No child living in household	5391	72.4%	3821	73.6%
Child living in household	2058	27.6%	1368	26.4%
Age of youngest child				
up to 5 years	457	6.1%	305	5.9%
6 to 10 years	327	4.4%	209	4.0%
11 to 17 years	533	7.2%	355	6.8%
Missing	741	9.9%	499	9.6%
**Employment status**				
Other work sector	2403	32.3%	2323	44.8%
Health and Education	1716	23.0%	443	8.5%
Retired	2115	28.4%	1637	31.5%
Unemployed	799	10.7%	484	9.3%
Missing	416	5.6%	302	5.8%
**Work in home office**				
No work in home office reported	2942	39.5%	1856	35.8%
Work at least partly in home office	1593	21.4%	1212	23.4%
Retired	2115	28.4%	1637	31.5%
Unemployed	799	10.7%	484	9.3%
**Monthly net household income**				
less than 2000 Euro	2818	37.8%	1354	26.1%
2000 to less than 6000 Euro	3379	45.4%	2832	54.6%

Attribute	Female subsample		Male subsample	
	N	%	N	%
6000 Euro or more	343	4.6%	414	8.0%
Missing	909	12.2%	589	11.4%
**Educational qualification**				
Academic education / University degree	2580	34.6%	2240	43.2%
Apprenticeship or Professional school diploma	1770	23.8%	934	18.0%
Qualification for university entrance	811	10.9%	454	8.7%
9 or 10 years of schooling	1711	23.0%	1117	21.5%
No professional qualification or still in training	50	0.7%	43	0.8%
Missing	527	7.1%	401	7.7%
**Contact to COVID-19 cases**				
No	4758	63.9%	3352	64.6%
Yes	960	12.9%	606	11.7%
Unclear contact	1731	23.2%	1231	23.7%
**Pre-existing health conditions**				
No pre-existing condition	4831	64.9%	3091	59.6%
Pre-existing condition	2576	34.6%	2068	39.9%
Unknown or not reported	42	0.6%	30	0.6%
**Smoking status**				
Never smoked	4543	61.0%	2537	48.9%
Ex-smoker	1826	24.5%	1785	34.4%
Smoker, occasionally or daily	1057	14.2%	846	16.3%
Invalid or missing response	23	0.3%	21	0.4%

[1] Single dose of Johnson&Johnson was considered as completely vaccinated
[2] Antibodies against spike, receptor-binding domain or nucleocapsid protein
[3] Based on self-reported vaccinations and infections

S5.2 Effect of seropositivity on contact numbers

Figure S5.2.1 Odds ratios for seropositivity due to infection in the MuSPAD gender subgroups. Results of the logistic regression models run for the exposure contact groups and the outcome seropositivity due to infection (s>1, rb>1, nc>1), separately for every contact setting in a) the female subsample and b) the male subsample, Odds ratios for seropositivity are given with 95% CI. For a list of adjustment variables for each model, refer to Table 5.2.1.

a. Female subsample

Outcome	Exposure	MuSPAD	OR (95% CI)
Seropositivity	Total contacts		
	up to 3 contacts		
	4-10 contacts		1.15 (0.90 to 1.46)
	11 or more contacts		1.35 (1.04 to 1.75)
Seropositivity	Household contacts		
	no household contact		
	1 household contact		1.58 (1.08 to 2.33)
	2 household contacts		1.24 (0.82 to 1.87)
	3 or more household contacts		1.62 (1.06 to 2.47)
Seropositivity	Non-household contacts		
	no non-household contact		
	1-3 non-household contacts		1.15 (0.84 to 1.58)
	4-10 non-household contacts		1.25 (0.91 to 1.72)
	11 or more non-household contacts		1.33 (0.96 to 1.85)

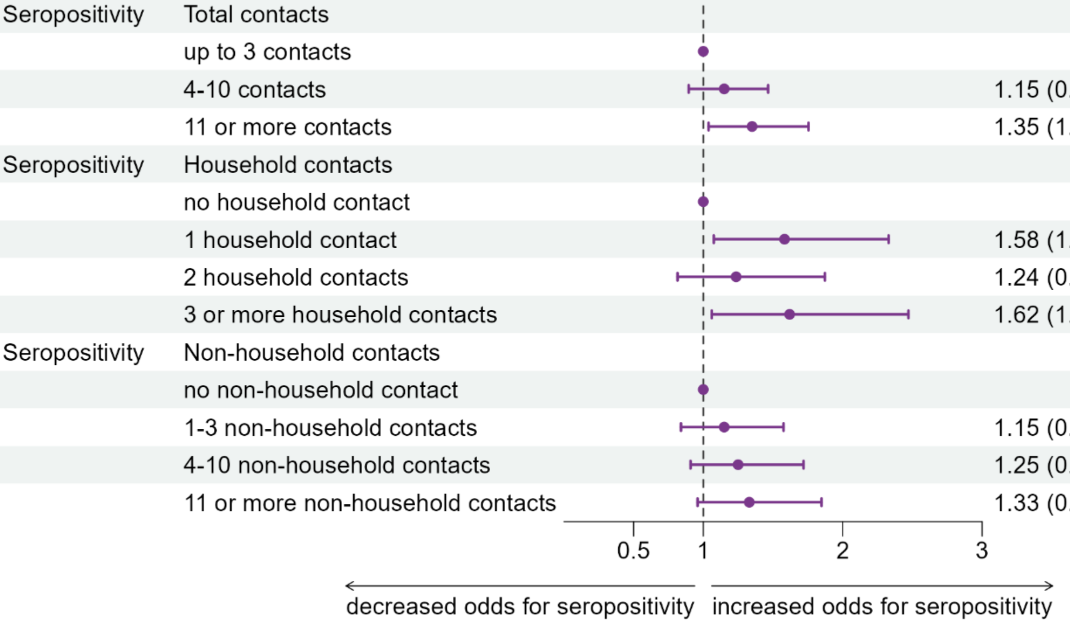

b. Male subsample

Outcome	Exposure	MuSPAD	OR (95% CI)
Seropositivity	Total contacts		
	up to 3 contacts		
	4-10 contacts		1.16 (0.87 to 1.55)
	11 or more contacts		1.24 (0.91 to 1.70)
Seropositivity	Household contacts		
	no household contact		
	1 household contact		1.25 (0.79 to 1.99)
	2 household contacts		1.14 (0.70 to 1.86)
	3 or more household contacts		1.53 (0.93 to 2.51)
Seropositivity	Non-household contacts		
	no non-household contact		
	1-3 non-household contacts		0.98 (0.67 to 1.43)
	4-10 non-household contacts		1.17 (0.80 to 1.71)
	11 or more non-household contacts		1.31 (0.89 to 1.92)

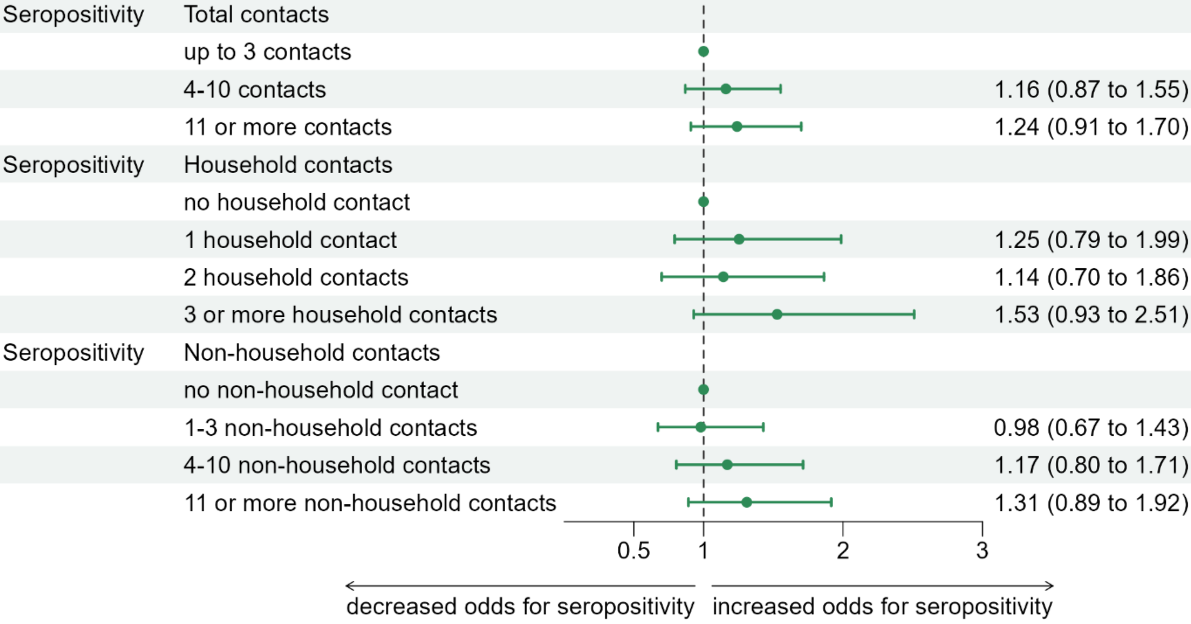

Table S5.2.1 Adjusted variables in gender-stratified logistic regression models for MuSPAD. List of which variables were used for each model given the restriction of different variables.

Model	MuSPAD adjusted variables
Logistic regression models for seropositivity due to infection for total contacts	Age,<br>Location (random effects),<br>Employment,<br>Working from home,<br>Monthly household income,<br>Socioeconomic status,<br>Child living in household,<br>Pre-existing condition,<br>7-day incidence,<br>Stringency index,<br>Vaccination status,<br>Smoking,<br>Vaccination proportion
Logistic regression models for seropositivity due to infection for household contacts	Age,<br>Location (random effects),<br>Employment,<br>Working from home,<br>Monthly household income,<br>Socioeconomic status,<br>Child living in household,<br>Vaccination proportion
Logistic regression models for seropositivity due to infection for non-household contacts	Age,<br>Location (random effects),<br>Employment,<br>Working from home,<br>Monthly household income,<br>Socioeconomic status,<br>Child living in household,<br>Pre-existing condition,<br>Household size,<br>7-day-incidence,<br>Stringency index,<br>Vaccination status,<br>Smoking,<br>Vaccination proportion

S5.3 Effect of children in the household on seropositivity

Figure S5.3.1 Odds ratios for seropositivity due to infection when living with a child in the MuSPAD gender subgroups.
Results of the logistic regression models run for the exposure living with a child in the household and the outcome seropositivity due to infection (s>1, rb>1, nc>1), separately for every contact setting in a) the female subsample and b) the male subsample, Odds ratios for seropositivity are given with 95% CI. For a list of adjustment variables for each model, refer to Table 5.3.1.

Outcome	Exposure	OR (95% CI)
Seropositivity	Female subset	
	No child living in household	
	Youngest child aged up to 5 years	1.32 (0.88 to 1.98)
	Youngest child aged 6 to 10 years	1.14 (0.72 to 1.81)
	Youngest child aged 11 to 17 years	1.29 (0.89 to 1.87)
	Living with child of unknown age	1.66 (1.17 to 2.34)
Seropositivity	Male subset	
	No child living in household	
	Youngest child aged up to 5 years	0.74 (0.45 to 1.22)
	Youngest child aged 6 to 10 years	1.11 (0.64 to 1.93)
	Youngest child aged 11 to 17 years	0.88 (0.55 to 1.41)
	Living with child of unknown age	0.72 (0.43 to 1.20)

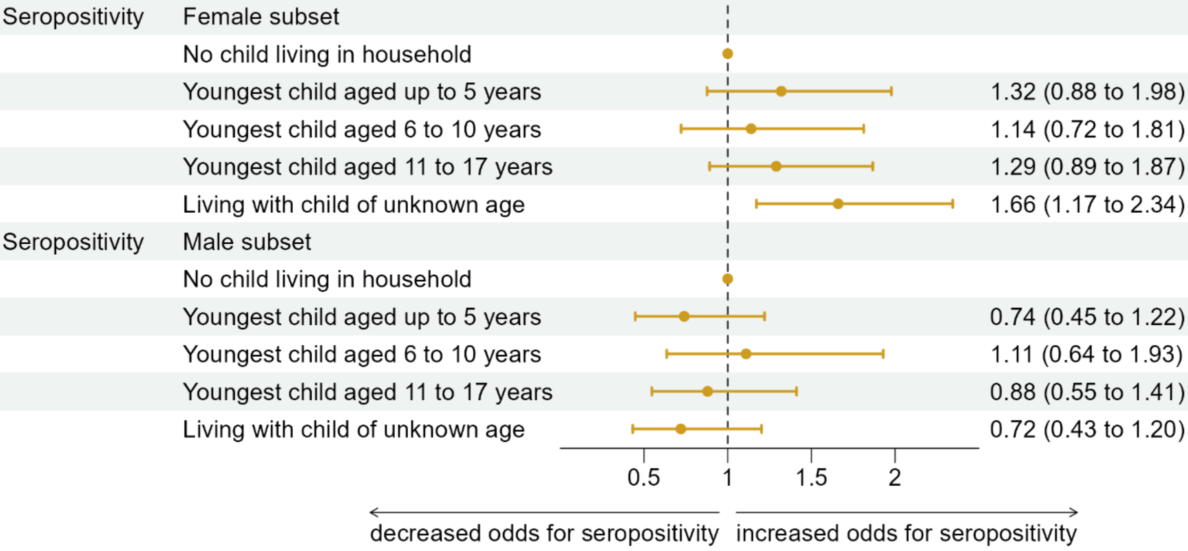

Table S5.3.1 Adjusted variables in gender-stratified logistic regression models for child in household for MuSPAD.
List of which variables were used for each model given the restriction of different variables.

Model	MuSPAD adjusted variables
Logistic regression models for seropositivity due to infection when living with a child	Age,<br>Location (random effects),<br>Employment,<br>Working from home,<br>Monthly household income,<br>Socioeconomic status,<br>Pre-existing condition,<br>Vaccination proportion,<br>Household density

S6 Serostatus and knowledge thereof

Figure S6.0.1 Forest plot of contact ratios by seropositivity and knowledge thereof. Contact ratios (CRs) using negative binomial regression models for total, household, and non-household contact numbers by seropositivity and knowledge thereof in MuSPAD. CRs for contacts are given with 95% confidence intervals. For a list of adjusted variables for each model, refer to Table 6.0.1.

Outcome	Exposure	CR (95% CI)
Total contacts		
	Seronegative and no knowledge of infection	
	Seropositive but no knowledge of infection	0.94 (0.85 to 1.03)
	Seropositive and knowledge of infection	1.08 (0.98 to 1.18)
Household contacts		
	Seronegative and no knowledge of infection	
	Seropositive but no knowledge of infection	1.07 (0.99 to 1.15)
	Seropositive and knowledge of infection	1.02 (0.96 to 1.09)
Non-household contacts		
	Seronegative and no knowledge of infection	
	Seropositive but no knowledge of infection	0.91 (0.78 to 1.06)
	Seropositive and knowledge of infection	1.14 (0.99 to 1.30)

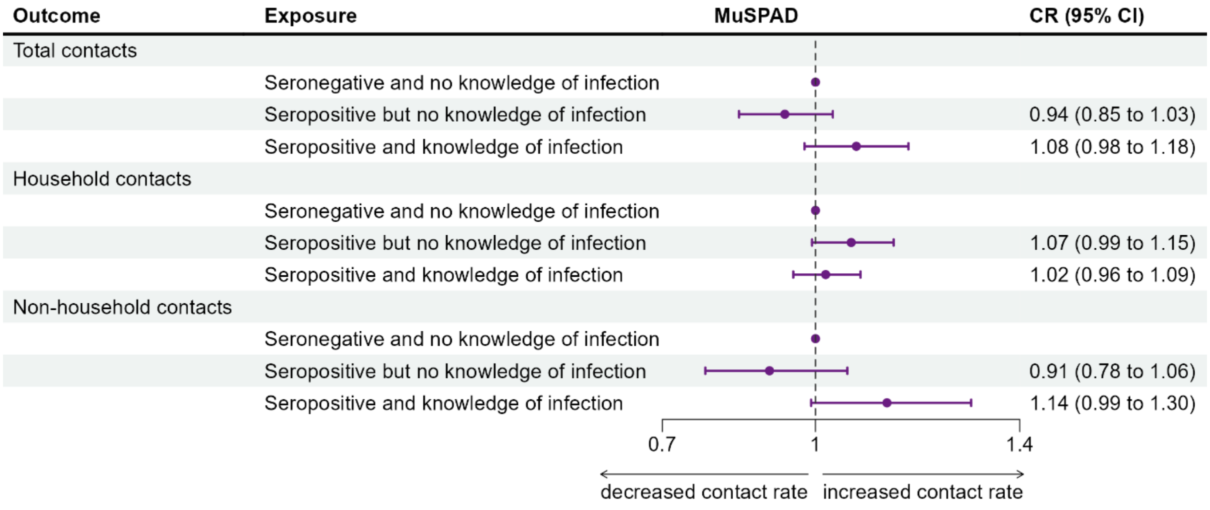

Table S6.0.1 Adjusted variables in sensitivity analysis regression models for MuSPAD.
List of which variables were used for each model given the restriction of different variables.

Model	MuSPAD adjusted variables
Negative binomial regression model for total contacts by serostatus and knowledge thereof	Age,<br>Gender,<br>Location (random effects),<br>Employment,<br>Working from home,<br>Monthly household income,<br>Socioeconomic status,<br>Child living in household,<br>Contact with infected individual,<br>COVID test status of household members,<br>Pre-existing condition,<br>7-day-incidence,<br>Stringency index,<br>Vaccination status,<br>Smoking,<br>Vaccination proportion
Negative binomial regression model for household contacts by serostatus and knowledge thereof	Age,<br>Gender,<br>Location (random effects),<br>Employment,<br>Working from home,<br>Monthly household income,<br>Socioeconomic status,<br>Child living in household,<br>Vaccination proportion
Negative binomial regression model for non-household contacts by serostatus and knowledge thereof	Age,<br>Gender,<br>Location (random effects),<br>Employment,<br>Working from home,<br>Monthly household income,<br>Socioeconomic status,<br>Child living in household,<br>Contact with infected individual,<br>COVID test status of household members,<br>Pre-existing condition,<br>Household size,<br>7-day-incidence,<br>Stringency index,<br>Vaccination status,<br>Smoking,<br>Vaccination proportion
